# Resources of Iranian agarics (Basidiomycota) with an outlook on their antioxidant potential

**DOI:** 10.3389/fmicb.2022.1015440

**Published:** 2022-10-28

**Authors:** Masoomeh Ghobad-Nejhad, Vladimír Antonín, Mohaddeseh Moghaddam, Ewald Langer

**Affiliations:** ^1^Department of Biotechnology, Iranian Research Organization for Science and Technology (IROST), Tehran, Iran; ^2^Department of Botany, Moravian Museum, Zelný trh 6, Brno, Czechia; ^3^Department of Ecology, University of Kassel, Kassel, Germany

**Keywords:** basidiomycetes, diversity, gilled mushrooms, ABTS assay, phylogeny

## Abstract

Agaric fungi are an important group of macromycetes with diverse ecological and functional properties, yet are poorly studied in many parts of the world. Here, we comprehensively analyzed 558 agaric species in Iran to reveal their resources of edible and poisonous species as well as their ecological guilds and luminescence potential. We also made a thorough survey of the antioxidant activity of the species. Phylogenetic relationships were reconstructed based on nuclear ribosomal LSU and ITS sequences. Our results reveal that agarics of Iran comprise about 189 edible, 128 poisonous, 254 soil saprotrophic, 172 ectomycorrhizal, 146 wood-inhabiting, 18 leaf/litter-inhabiting, 9 parasitic, and 19 luminescent species. Twenty percent of the Iranian agaric species possess antioxidant activity, phylogenetically distributed in four orders and 21 agaric families. About 5% of the antioxidant species can be considered strong antioxidants, many of which are also edible and could be utilized to develop functional foods. This is the first study combining phylogeny and antioxidant potential of agaric mushrooms in a large scale, and the obtained results would guide the selection of agaric taxa to be examined in the future for taxonomic revisions, biotechnological applications, and applied phylogeny studies.

## Introduction

Agarics are mushroom-forming fungi also called euagarics and their hymenium is formed on gills. They belong to the subdivision Agaricomycotina, class Agaricomycetes (Moncalvo et al., 2002; Bauer et al., 2006). They produce important natural substances used in agriculture (e.g., strobilurines), medicine (e.g., pleuromutilines), and biotechnology (e.g., polysaccharides; Pointing et al., 2001; Webster and Weber, 2007; Kück et al., 2014; Hyde et al., 2019; Sandargo et al., 2019). Agaricales is the largest fungal order of agaric mushrooms comprising *ca.* 13,000 known species (Kirk et al., 2008). Thorough investigations of the agarics phylogeny have recently been provided by He et al. (2019) and Varga et al. (2019). Some agarics are important model organisms for research in genetics and basidiome development such as *Coprinopsis cinerea* and *Cyclocybe cylindrica* (Herzog et al., 2019). Among agarics, there are some of the most poisonous mushrooms such as *Amanita phalloides*, *Cortinarius* Subgen. *Orellani*, and *Inosperma erubescens*, frequently mixed up with edible mushrooms during culinary collecting and thus causing severe fatalities. Nevertheless, there is a large number of edible agaric mushrooms highly prized for culinary purposes such as *Agaricus campestris*, *Coprinus comatus*, *Cyclocybe cylindrica*, *Macrolepiota procera*, and the worldwide cultivated white button mushroom *Agaricus bisporus*. Several edible agaric species are saprotrophs and possible to cultivate, but there are also many edible species such as *Russula* spp. and *Lactarius* spp. which belong to the ectomycorrhizal ecological guild and thus not cultivable in artificial synthetic media. A number of species such as *Lentinula edodes* and *Flammulina velutipes* have culminated as functional mushrooms for developing mushroom-based functional foods and other valued mycochemicals (Chang, 1996; Cateni et al., 2022; Rodríguez-Seoane et al., 2022).

Numerous agarics have also been recognized as sources of antioxidant compounds (e.g., Ferreira et al., 2009; Asatiani et al., 2010; Guo et al., 2012; Wang and Xu, 2014; Sánchez, 2017; Islam et al., 2019; Thu et al., 2020). Antioxidant properties, or the ability to defend against and scavenge/reduce excess free radicals in biological systems, is among the important properties of living organisms and crucial for their survival (Xiao et al., 2020). Mushrooms as one of the most diverse natural antioxidant resources, have received attention in recent decades and are advantageous compared to plants because of their high diversity, fast growth, and culture possibilities (Gargano et al., 2017; Buswell, 2018).

A preliminary checklist of Iranian mushrooms appeared by Ghobad-Nejhad et al. (2020) listing 556 agaric and 29 bolete species. However, the species remain largely unexplored in terms of various important properties. Information about the edible, poisonous, and mycorrhizal agarics in Iran is principally lacking and currently, the antioxidant properties of Iranian agarics have remained largely unexplored.

Due to the lack of knowledge about the diversity of edible, poisonous, and mycorrhizal agarics in Iran, as well as their antioxidant properties, our study aimed to: (i) investigate Iranian agarics and reveal their resources of edible and poisonous species, (ii) present their ecological guilds and bioluminescence potential, and to (iii) explore the antioxidant properties of Iranian agarics and combine it with phylogenetic reconstructions. We believe our results would benefit a wide range of researchers involved in the study of agaric mushrooms.

## Materials and methods

### Sampling and molecular study

Taxon sampling for the molecular study was primarily done based on the list by Ghobad-Nejhad et al. (2020), supplemented by additional data in the present study. Species current names and species authorities follow Index Fungorum^1^ and MycoBank.^2^ Microscopy and morphological studies followed Ghobad-Nejhad et al. (2020). Sequences of the 28S rRNA (nLSU) and the ITS region (covering ITS1, 5.8, and ITS2) were carefully selected from GenBank, with special attention to the quality-controlled sequences (Nilsson et al., 2012) as well as to the authentic sequences obtained from Iranian specimens. For DNA extraction, we sampled more than 20 specimens and 12 samples were successfully sequenced and used in this paper. Genomic DNA was extracted from dried basidiomata using the DNA Extraction Mini Kit (FAVORGEN, Taiwan). The primers used for the amplification cycles were ITS1F/ITS4B or ITS1F/ITS4 (White et al., 1990; Gardes and Bruns, 1993) for the ITS region and LR0R/LR7 or LR0R/LR5 (Hopple and Vilgalys, 1999) for partial nLSU region. All sequences used in the phylogenetic analyses are listed in Table 1.

**Table 1:** Resources of agarics of Iran and their edibility (☺, edible; ☺, edible based on own observation in Iran; ☹, poisonous; ☹, poisonous based on own observation in Iran; ☺*, edible if well-cooked but poisonous if raw; X, inedible; ○, uncertain or unknown), ecological guild (♠, soil saprotroph; ☼, ectomycorrhizal; ▐, wood-inhabiting; ♣, leaf/litter-inhabiting; ◙, parasitic), luminescence, and antioxidant potential (S, strong; M, moderate; W, weak; ND, not determined; full details provided in the text).

Species	Edibility	Ecological guild	Luminescent	Antioxidant potential	GenBank accession no.	
					ITS	nLSU
*Agaricus arvensis* Schaeff.	☺	♠	–	S	MT535720	MH872779
*Agaricus bisporus* (J.E. Lange) Imbach	☺	♠	–	SM	**ON952490**	DQ071710
*Agaricus bitorquis* (Quél.) Sacc.	☺	♠	–	SM	MT535709	MT554302
*Agaricus bresadolanus* Bohus	☺	♠	–	MW	DQ185569	MK277477
*Agaricus brunneolus* (J.E. Lange) Pilát	☺	♠	–	ND	KU975082	KX083997
*Agaricus campestris* L.	☺	♠	–	SM	NR_151745.1	MH868030
*Agaricus depauperatus* (F.H. Møller) Pilát	☺	♠	–	ND	DQ182530	–
*Agaricus devoniensis* P.D. Orton	☺	♠	–	ND	EU363036	AF059225
*Agaricus gennadii* (Chatin & Boud.) P.D. Orton	☺	♠	–	ND	KT951318	KR006606
*Agaricus iodosmus* Heinem.	☹	♠	–	S	MT535702	MT554295
*Agaricus iranicus* Mahdizadeh, Safaie, Goltapeh, L.A. Parra & Callac	○	♠	–	ND	KY474556	KY474559
*Agaricus langei* (F.H. Møller) F.H. Møller	☺	♠	–	ND	JF797181	–
*Agaricus litoralis* (Wakef. & A. Pearson) Pilát	☺	♠	–	ND	MT535711	MT554304
*Agaricus moelleri* Wasser	☹	♠	–	ND	KT824787	–
*Agaricus nevoi* Wasser	○	♠	–	ND	MH173866	–
*Agaricus phaeolepidotus* (F.H. Møller) F.H. Møller	☹	♠	–	ND	MH862921	MH874494
*Agaricus pseudolutosus* (G. Moreno, Esteve-Rav., Illana & Heykoop) G. Moreno, L.A. Parra, Esteve-Rav. & Heykoop	☺	♠	–	ND	KT951329	KT951453
*Agaricus pseudopratensis* (Bohus) Wasser	☹	♠	–	S	**ON952491**	MT554325
*Agaricus subrufescens* Peck	☺	♠	–	M	KT983412	KT951461
*Agaricus xanthodermus* Genev.	☹	♠	–	ND	KT824789	KR006612
*Agrocybe acericola* (Peck) Singer	○	♠	–	ND	MN860126	MK277500
*Agrocybe dura* (Bolton) Singer	☺	♠ ▐ (saprothrophic on soil, but on decaying woody remnants)	–	S	MT535714	MT554306
*Agrocybe ochracea* Nauta	X	♠ ▐ (saprothrophic on soil, but on decaying woody remnants)	–	ND	–	–
*Agrocybe paludosa* (J.E. Lange) Kühner & Romagn. ex Bon	X	♠	–	ND	–	–
*Agrocybe pediades* (Fr.) Fayo	X	♠	–	M	**ON952487, ON952488**	AY293582
*Agrocybe praecox* (Pers.) Fayod	☺	♠ ▐ (saprothrophic on soil, on decaying woody remnants)	–	W	MT535701	MT554294
*Agrocybe pusiola* (Fr.) R. Heim	X	♠	–	ND	DQ389732	MK277505
*Agrocybe tabacina* (DC.) Konrad & Maubl.	X	♠	–	ND	–	–
*Agrocybe vervacti* (Fr.) Singer	X	♠	–	ND	MW425942	MK277506
*Alnicola escharioides* (Fr.) Romagn.	X	☼	–	ND	MW243076	–
*Amanita atkinsoniana* Coker	○	☼	–	ND	MZ668014	MK277560
*Amanita battarrae* (Boud.) Bon	☺*	☼	–	ND	MH508267	MH486389
*Amanita caesarea* (Scop.) Pers.	☺	☼	–	M	MZ005548	AF024443
*Amanita ceciliae* (Berk. & Broome) Bas	☺*	☼	–	ND	OK299150	OK299170
*Amanita crocea* (Quél.) Singer	☺*	☼	–	M	KJ638266	–
*Amanita eliae* Quél.	☹	☼	–	ND	KF780872	–
*Amanita excelsa* (Fr.) Bertill.	☺	☼	–	ND	MW258873	MW258922
*Amanita gemmata* (Fr.) Bertill.	☹	☼	–	ND	MK580689	AF024457
*Amanita lividopallescens* (Secr. ex Boud.) Kühner & Romagn. agg.	☺ ☹*	☼	–	ND	MT535691	MW013165
*Amanita pantherina* (DC.) Krombh.	☹	☼	–	M	FR852274	MH486743
*Amanita phalloides* (Fr.) Link	☹	☼	–	ND	KX449212	KX449230
*Amanita rubescens* Pers.	☺	☼	–	S	FR852273	MH486816
*Amanita strobiliformis* (Paulet ex Vittad.) Bertill.	☹ X	☼	–	ND	MH508614	MH486895
*Amanita umbrinolutea* (Secr. ex Gillet) Bataille	☺*	☼	–	ND	MH508641	MH486937
*Amanita vaginata* (Bull.) Lam. s.l.	☺*	☼	–	M	JF907756	–
*Amanita verna* (Bull.) Lam.	☹	☼	–	ND	EU909448	HQ539755
*Ampulloclitocybe clavipes* (Pers.) Redhead, Lutzoni, Moncalvo, and Vilgalys	☺ ☹ (seems to be toxic after consumption with alcohol)	♠	–	ND	AY789080	AY639881
*Armillaria borealis* Marxm. & Korhonen	☺*	♠ ◙	Desjardin et al. (2008)	ND	KP960524	FJ618728
*Armillaria cepistipes* Velen.	☺*	♠ ◙	Mihail (2015)	ND	FJ903313	KY418876
*Armillaria gallica* Marxm. & Romagn.	☺*	♠ ◙	Kotlobay et al. (2018)	ND	MW418538	AM269818
*Armillaria mellea* (Vahl) P. Kumm.	☺*	♠ ◙	Kotlobay et al. (2018)	SM	AF163583	AM269819
*Arrhenia griseopallida* (Desm.) Watling	X	♠	–	ND	–	–
*Asterophora lycoperdoides* (Bull.) Ditmar	X	◙	–	ND	MZ159455	MK277604
*Atheniella flavoalba* (Fr.) Redhead, Moncalvo, Vilgalys, Desjardin, and B.A. Perry	X	♠	–	ND	MH857185	MH868723
*Baeospora myosura* (Fr.) Singer	X	♠ (on conifer cones)	–	ND	MH856301	MH867849
*Battarrea stevenii* (Libosch.) Fr.	X	♠	–	ND	AF215648	–
*Bolbitius reticulatus* (Pers.) Ricken	X	▐	–	ND	JX968249	JX968366
*Bolbitius titubans* (Bull.) Fr.	X ☹	♠	–	ND	KR425522	KR425552
*Calocybe carnea* (Bull.) Donk	☺	♠	–	ND	AF357028	MK277666
*Calocybe chrysenteron* (Bull.) Singer	X	♠	–	ND	KP885639	KP885628
*Calocybe gambosa* (Fr.) Donk	☺	♠	–	W	MZ159691	AM946414
*Calocybe ionides* (Bull.) Donk	☺	♠	–	ND	JF907780	MK277668
*Calocybe persicolor* (Fr.) Singer	X ☺ ☹ (edible in the Czech Republic)	♠	–	ND	KP192564	AF223176
*Candolleomyces candolleanus* (Fr.) D. Wächt. & A. Melzer		▐	–	S	MT535718	MT554309
*Cantharellus alborufescens* (Malençon) Papetti & S. Alberti	☺	☼	–	M	MH463257	MH463258
*Cantharellus cibarius* Fr.	☺	☼	–	SM	KX907204	KX828805
*Cantharellus ferruginascens* P.D. Orton	☺	☼	–	ND	MH463294	MH463295
*Chlorophyllum brunneum* (Farl. & Burt) Vellinga	☹	♠	–	ND	MG742013	MG742022
*Chlorophyllum rhacodes* (Vittad.) Vellinga	☺	♠	–	M	AY081236	AY176345
*Clitocybe angustissima* (Lasch) P. Kumm.	☹	♠	–	ND	–	–
*Clitocybe barbularum* (Romagn.) P.D. Orton	X	♠	–	ND	–	–
*Clitocybe diatreta* (Fr.) P. Kumm.	☹	♠	–	ND	–	–
*Clitocybe metachroa* (Fr.) P. Kumm.	X	♠	–	ND	JF907806	AY207155
*Clitocybe nebularis* (Batsch) P. Kumm.	☺	♠	–	S	DQ149727	AY586685
*Clitocybe phyllophila* (Pers.) P. Kumm.	☹	♠	–	ND	MH856300	MH867847
*Clitocybe rufuloalutacea* Métrod ex Bon	○	♠	–	ND	–	–
*Clitocybe vibecina* (Fr.) Quél.	X	♠	–	ND	JF907821	AY207160
*Clitopaxillus alexandri* (Gillet) G. Moreno, Vizzini, Consiglio & P. Alvarado	☺	♠	–	W	MG321345	MG321393
*Clitopilus prunulus* (Scop.) P. Kumm.	☺	♠ ☼	–	M	FJ770408	GU384615
*Clitopilus scyphoides* (Fr.) Singer	X	♠	–	ND	MH856181	MH867707
*Collybia tuberosa* (Bull.) P. Kumm.	X	♠ ◙	Malakauskienė (2018)	ND	AY854072	AY639884
*Conocybe albipes* (G.H. Otth) Hauskn.	X	♠	–	ND	–	–
*Conocybe apala* (Fr.) Arnolds	☹	♠	–	ND	MT535728	MT554318
*Conocybe dunensis* T.J. Wallace	X	♠	–	ND	JX968227	JX968345
*Conocybe juniana* (Velen.) Hauskn. & Svrček	X	♠	–	ND	JX968191	JX968307
*Conocybe leucopus* Kühner ex Kühner & Watling	X	♠	–	ND	–	–
*Conocybe macrocephala* Kühner & Watling	X	♠	–	ND	JX968182	JX968298
*Conocybe microspora* (Velen.) Dennis	X	♠	–	ND	JX968160	JX968276
*Conocybe ochracea* Kühner ex Singer	X	♠	–	ND	–	–
*Conocybe olivaceopileata* E.F. Malysheva^a^	X	♠	–	ND	**ON952486**	–
*Conocybe pilosella* (Pers.) Kühner	X	♠	–	ND	JX968231	JX968349
*Conocybe rickenii* (Jul. Schäff.) Kühner	X	♠	–	ND	AY194541	AY293597
*Conocybe subovalis* Kühner & Watling	X	♠	–	ND	JX968190	JX968306
*Conocybe tenera* (Schaeff.) Fayod	☹	♠	–	M	MH855754	MH867266
*Contumyces rosellus* (M.M. Moser) Redhead, Moncalvo, Vilgalys, and Lutzoni	○	♠	–	ND	OL771755	OL771796
*Coprinellus angulatus* (Peck) Redhead	X	♠ ▐ (on burnt ground/wood)	–	ND	MN121285	MH868315
*Coprinellus disseminatus* (Pers.) J.E. Lange	X	▐	–	ND	MK050584	AY207180
*Coprinellus domesticus* (Bolton) Vilgalys, Hopple & Jacq. Johnson	X ☹	▐	–	S	MH856480	MH868019
*Coprinellus flocculosus* (DC.) Vilgalys, Hopple & Jacq. Johnson	X	♠ ▐	–	ND	FN396138	FN396208
*Coprinellus impatiens* (Fr.) J.E. Lange	X	♠	–	ND	MH856810	MH868327
*Coprinellus micaceus* (Bull.) Vilgalys, Hopple & Jacq. Johnson	X ☹	▐	–	S	**ON952489**	MT554289
*Coprinellus radians* (Desm.) Vilgalys, Hopple & Jacq. Johnson	X	▐	–	ND	KU375662	KM272009
*Coprinellus silvaticus* (Peck) Gminder	X	♠ ▐	–	ND	HQ846986	HQ847072
*Coprinellus subimpatiens* (M. Lange & A.H. Sm.) Redhead, Vilgalys, and Moncalvo	X	♠ ▐	–	ND	MH857001	MH868522
*Coprinellus truncorum* (Scop.) Redhead, Vilgalys, and Moncalvo	X	▐	–	S	FM878007	FM876263
*Coprinellus xanthothrix* (Romagn.) Vilgalys, Hopple & Jacq. Johnson	X	▐	–	ND	JN943112	JN159595
*Coprinopsis atramentaria* (Bull.) Redhead, Vilgalys, and Moncalvo	☺ ☹ (toxic after consumption with alcohol)	▐	–	SM	MH259864	FN396172
*Coprinopsis brunneofibrillosa* (Dennis) Redhead, Vilgalys, and Moncalvo	X	▐	–	ND	JX118664	JX118817
*Coprinopsis cinerea* (Schaeff.) Redhead, Vilgalys, and Moncalvo	X	♠	–	M	MF161131	KM272007
*Coprinopsis ephemeroides* (DC.) G. Moreno	X	♠	–	ND	–	–
*Coprinopsis friesii* (Quél.) P. Karst.	X	♣	–	ND	–	FN396191
*Coprinopsis gonophylla* (Quél.) Redhead, Vilgalys & Moncalvo	X	♠	–	ND	MH856188	MH867714
*Coprinopsis lagopides* (P. Karst.) Redhead, Vilgalys & Moncalvo	X	♠	–	ND	MN892574	AF041488
*Coprinopsis lagopus* (Fr.) Redhead, Vilgalys & Moncalvo	X	♠ ▐ ♣	–	ND	MH856194	MH867720
*Coprinopsis macrocephala* (Berk.) Redhead, Vilgalys & Moncalvo	X	♠ ♣	–	ND	FN396126	FN396175
*Coprinopsis marcescibilis* (Britzelm.) Örstadius & E. Larss.	X	♠ ▐	–	ND	**ON952484**	FM876278
*Coprinopsis martinii* (P.D. Orton) Redhead, Vilgalys & Moncalvo	X	♣	–	ND	GU234126	–
*Coprinopsis nivea* (Pers.) Redhead, Vilgalys & Moncalvo	X	♠	–	ND	HQ847032	HQ847117
*Coprinopsis patouillardii* (Quél.) Gminder	X	♠	–	ND	FN396150	FN396197
*Coprinopsis picacea* (Bull.) Redhead, Vilgalys & Moncalvo	X ☹	♠	–	S	JN943110	JQ045885
*Coprinopsis sclerotiger* (Watling) Redhead, Vilgalys & Moncalvo	X	♠	–	ND	MF161091	MF161132
*Coprinopsis scobicola* (P.D. Orton) Redhead, Vilgalys & Moncalvo	X	♠ ▐	–	ND	HQ847021	HQ847106
*Coprinopsis urticicola* (Berk. & Broome) Redhead, Vilgalys & Moncalvo	X	▐ ♣	–	ND	MH300615	HQ847101
*Coprinus comatus* (O.F. Müll.) Pers.	☺ (considered edible in Europe and also cultivated)	♠	–	SM	MH817141	MH997559
*Coprinus sterquilinus* (Fr.) Fr.	X	♠	–	ND	MH854689	AF041530
*Cortinarius bivelus* (Fr.) Fr.	X	☼	–	ND	FR852016	–
*Cortinarius caesiocortinatus* Jul. Schäff.	X	☼	–	ND	FR852020	–
*Cortinarius casimirii* (Velen.) Huijsman	X	☼	–	ND	FR851999	–
*Cortinarius causticus* Fr.	X	☼	–	ND	FJ157016	FJ157016
*Cortinarius cinnabarinus* Fr.	X	☼	–	ND	KC842405	KC842476
*Cortinarius cinnamomeus* (L.) Gray	☹	☼	–	ND	NR_131816	KC842483
*Cortinarius cotoneus* Fr.	☺ X	☼	–	ND	KC842423	KC842493
*Cortinarius decipiens* Fr.	X	☼	–	ND	HE687043	–
*Cortinarius diasemospermus* Lamoure	X	☼	–	ND	HE687042	–
*Cortinarius erumpens* Rob. Henry	○	☼	–	ND	–	–
*Cortinarius ferrugineovelatus* Kytöv., Liimat. & Niskanen	○	☼	–	ND	NR_131875	MK277631
*Cortinarius fluryi* (M.M. Moser) M.M. Moser	○	☼	–	ND	–	–
*Cortinarius hildegardiae* Schmidt-Stohn, Brandrud & Dima	○	☼	–	ND	MT535704	MT554297
*Cortinarius hinnuleus* Fr.	X	☼	–	ND	AY083183	AF388779
*Cortinarius infractus* (Pers.) Fr.	X	☼	–	ND	NR_130225	KC842497
*Cortinarius olivaceofuscus* Kühner	☹	☼	–	ND	AY669585	MK277762
*Cortinarius paracephalixus* Bohus	X	☼	–	ND	KR080708	–
*Cortinarius parvannulatus* Kühner	X	☼	–	ND	HE687041	–
*Cortinarius persoonianus* Bidaud	○	☼	–	S	MT535741	MT554330
*Cortinarius pluviorum* Jul. Schäff. ex M.M. Moser	X	☼	–	ND	FJ157038	FJ157038
*Cortinarius uraceonemoralis* Niskanen, Liimat., Dima, Kytöv., Bojantchev & H. Lindstr.	○	☼	–	ND	NR_131836	MK277663
*Cortinarius valgus* Fr.	X	☼	–	ND	MT935583	–
*Cortinarius vernus* H. Lindstr. & Melot	X	☼	–	ND	MW263848	MW263545
*Cortinarius vespertinus* (Fr.) Fr.	X	☼	–	ND	KC842457	KC842527
*Cortinarius vibratilis* (Fr.) Fr.	X ☹	☼	–	ND	KC842440	KC842510
*Cortinarius violaceus* (L.) Gray	☺	☼	–	W	NR_173726	MK277758
*Craterellus cinereus* (Pers.: Fr.) Maire	☺	☼	–	ND	–	–
*Craterellus cornucopioides* (L.) Pers.	☺	☼	–	SM	JF907967	MN227282
*Craterellus tubaeformis* (Fr.) Quél.	☺	☼	–	S	HM468493	MF797698
*Crepidotus applanatus* (Pers.) P. Kumm.	X	▐	–	ND	MH855941	MH867439
*Crepidotus caspari* Velen.	X	▐	–	ND	MW722982	AF205678
*Crepidotus cesatii* (Rabenh.) Sacc.	X	▐	–	ND	JF907962	MK277881
*Crepidotus crocophyllus* (Berk.) Sacc.	X	▐	–	ND	FJ596825	AF367939
*Crepidotus mollis* (Schaeff.) Staude	X	▐	–	ND	AM882996	AM882996
*Crepidotus subverrucisporus* Pilát	X	▐	–	ND	MT535745	AF367948
*Crinipellis scabella* (Alb. & Schwein.) Murrill	X	♠	–	ND	MH857177	MH868716
*Cuphophyllus virgineus* (Wulfen) Kovalenko	☺	♠	–	ND	MT535688	MT554284
*Cyclocybe cylindracea* (DC.) Vizzini & Angelini	☺	▐	–	MW	**ON952480, ON952485**	**ON930146**
*Cystoderma aureum* (Matt.) Kühner & Romagn.	☺ X (generally edible, but some health problems described after eating)	♠	–	ND	MH864957	MH876401
*Deconica coprophila* (Bull.) P. Karst.	☹ X	♠	–	ND	MH855878	MH867388
*Deconica crobula* (Fr.) Romagn.	X	♠	–	ND	MT535747	MH867478
*Delicatula integrella* (Pers.) Fayod	X	▐	–	ND	MZ159362	MK277924
*Dermoloma cuneifolium* (Fr.) Singer ex Bon	X	♠	–	ND	MW193843	–
*Echinoderma asperum* (Pers.) Bon	☹	♠	–	W	MH856136	MH867652
*Entoloma clypeatum* (L.) P. Kumm.	☺ (frequently eaten in the Czech Republic, considered poisonous in China^b^)	☼	–	ND	KC710059	KC710136
*Entoloma griseoluridum* (Kühner) M.M. Moser	X	♠	–	ND	–	–
*Entoloma griseorubellum* (Lasch) Kalamees & Urbonas	○	♠	–	ND	–	–
*Entoloma hirtipes* (Schumach.) M.M. Moser	X	♠	–	ND	MN088710	MN088715
*Entoloma incanum* (Fr.) Hesler	☹ X	♠	–	ND	OK161249	OK161276
*Entoloma majaloides* P.D. Orton	X	♠	–	ND	MW633049	MW633049
*Entoloma mammosum* (L.) Hesler	○	♠	–	ND	–	–
*Entoloma niphoides* Noordel.	X	☼	–	ND	JF907999	FJ794075
*Entoloma rhodopolium* (Fr.) P. Kumm.	☹	♠	–	ND	LN850497	LN850705
*Entoloma sericellum* (Fr.) P. Kumm.	X	♠	–	ND	KC898453	GQ289190
*Entoloma sinuatum* (Bull. ex Pers.) P. Kumm.	☹	☼	–	ND	KC710116	KC710154
*Entoloma subcollariatum* (Kühner) Bon	○	♠	–	ND	MH453494	–
*Entoloma vernum* S. Lundell	☹	♠	–	ND	MF476911	MF487802
*Flammula alnicola* (Fr.) P. Kumm.	☺ ☹ (considered edible in the Czech Republic)	♠	–	ND	MH862103	MH873792
*Flammulaster erinaceellus* (Peck) Watling	X	▐	–	ND	MF755278	EF537889
*Flammulaster ferrugineus* (Maire) Watling	X	▐	–	ND	MF039253	–
*Flammulaster gracilis* (Quél.) Watling	X	▐	–	ND	–	–
*Flammulaster granulosus* (J.E. Lange) Watling	X	♠	–	ND	–	–
*Flammulina velutipes* (Curtis) Singer	☺	▐	Desjardin et al. (2008)	SM	MT535715	MT554307
*Galerina hypnorum* (Schrank) Kühner	☹	♠	–	ND	OL771728	MK299406
*Galerina marginata* (Batsch) Kühner	☹	▐	–	ND	MK346203	MK346279
*Galerina mniophila* (Lasch) Kühner	X	♠	–	ND	AJ585456	AJ871514
*Galerina pumila* (Pers.) M. Lange	X	♠	–	ND	AJ585477	AJ871546
*Galerina sphagnorum* (Pers.) Kühner	X	♠	–	ND	AJ585455	AJ871510
*Gymnopilus penetrans* (Fr.) Murrill	☹ X (considered inedible in the Czech Republic)	▐	–	W	KR011987	KR011988
*Gymnopilus spectabilis* (Weinm.) A.H. Sm.	☹ X (considered inedible in the Czech Republic)	▐	–	S	MT535703	MT554296
*Gymnopus androsaceus* (L.) J.L. Mata & R.H. Petersen	X	▐ ♣	–	ND	MH857176	MH868715
*Gymnopus aquosus* (Bull.) Antonín & Noordel.	☺	♠	–	ND	MT535700	MT554293
*Gymnopus brassicolens* (Romagn.) Antonín & Noordel.	X	▐ ♠	–	ND	MZ088117	MK278106
*Gymnopus dryophilus* (Bull.) Murrill	☺ (considered poisonous in China)	♠	–	W	MH589967	MH589985
*Gymnopus erythropus* (Pers.) Antonín, Halling & Noordel.	☺	▐	–	ND	JX536136	AY207167
*Gymnopus foetidus* (Sowerby) J.L. Mata & R.H. Petersen	X	▐	–	ND	KY026682	KY026682
*Gymnopus fusipes* (Bull.) Gray	☺ X (only young basidiomata edible)	▐ ◙	–	W	KY026727	KY026727
*Gymnopus hybridus* (Kühner & Romagn.) Antonín & Noordel.	X	♠	–	ND	MT535705	MT554299
*Gymnopus inodorus* (Pat.) Antonín & Noordel.	X	▐	–	ND	–	–
*Gymnopus terginus* (Fr.) Antonín & Noordel.	X	♠	–	ND	–	MK278118
*Hebeloma birrus* (Fr.) Gillet	X	☼	–	ND	JF908029	–
*Hebeloma crustuliniforme* (Bull.) Quél.	☹	☼	–	ND	MH856151	MH867674
*Hebeloma hiemale* Bres.	X	☼	–	ND	KT591536	KT591556
*Hebeloma incarnatulum* A.H. Sm.	X	☼	–	ND	KX687211	–
*Hebeloma mesophaeum* (Pers.) Quél.	X	☼	–	ND	NR_173705	MK880553
*Hebeloma sinapizans* (Paulet) Gillet	☹	☼	–	M	KT591542	KT591562
*Hemimycena cucullata* (Pers.) Singer	X	▐ ♠ (most frequently wood-inhabiting)	–	ND	–	–
*Hodophilus hymenocephalus* (A.H. Sm. & Hesler) Birkebak & Adamčík	X	♠	–	ND	DQ484066	DQ457679
*Hohenbuehelia atrocoerulea* (Fr.) Singer	X	▐	–	ND	KU355304	KU355389
*Hohenbuehelia auriscalpium* (Maire) Singer	X	▐	–	ND	MT525860	MT534052
*Hohenbuehelia petaloides* (Bull.) Schulzer	☺	▐	–	ND	NR_173155	KU355402
*Homophron spadiceum* (P. Kumm.) Örstadius & E. Larss.	X	▐	–	ND	MK968340	MN028523
*Hydropus marginellus* (Pers.) Singer	X	▐	–	ND	DQ490627	DQ457674
*Hygrocybe acutoconica* (Clem.) Singer	☹	♠	–	ND	OK157438	MK278174
*Hygrocybe chlorophana* (Fr.) Wünsche	☺	♠	–	ND	JF908052	MK278164
*Hygrophorus eburneus* (Bull.) Fr.	☺	☼	–	S	MK088116	AF430279
*Hygrophorus mesotephrus* Berk. & Broome	☺	☼	–	ND	MT981695	–
*Hygrophorus persoonii* Arnolds	☺	☼	–	ND	MN243172	KF291213
*Hymenopellis radicata* (Relhan) R.H. Petersen	☺	▐	–	M	MZ159452	MT554280
*Hypholoma capnoides* (Fr.) P. Kumm.	☺	▐	–	W	FJ596780	AY207211
*Hypholoma fasciculare* (Huds.) P. Kumm.	☹	▐	–	SM	MT535706	MT554300
*Hypholoma lateritium* (Schaeff.) P. Kumm.	☹ X	▐	–	SM	MH856121	MH866989
*Hypholoma radicosum* J.E. Lange	X (considered edible in China)	▐	–	ND	–	DQ071685
*Hypholoma subericaeum* (Fr.) Kühner	X	♠	–	ND	–	MK278215
*Hypsizygus ulmarius* (Bull.) Redhead	☺	▐	–	S	AY265850	AF042584
*Infundibulicybe geotropa* (Bull. ex DC.) Harmaja	☺ (considered poisonous in China)	♠	–	MW	KT122792	KT122793
*Infundibulicybe gibba* (Pers.) Harmaja	☺	♠	–	ND	MH856103	MZ719010
*Infundibulicybe trulliformis* (Fr.) Gminder	X	♠	–	ND	JF907809	–
*Inocybe amethystina* Kuyper	☹	☼	–	ND	HE687066	–
*Inocybe asterospora* Quél.	☹	☼	–	ND	HM060326	HM060325
*Inocybe castaneicolor* A. La Rosa, Bizio, Saitta & Tedersoo	☹	☼	–	ND	KY213954	KY213954
*Inocybe cincinnata* (Fr.) Quél.	☹	☼	–	ND	MG489949	KC305372
*Inocybe corydalina* Quél.	☹	☼	–	ND	MH216083	MH220259
*Inocybe decemgibbosa* (Kühner) Vauras	☹	☼	–	ND	HE687073	–
*Inocybe flocculosa* Sacc.	☹	☼	–	ND	LT716045	KY418861
*Inocybe geophylla* (Sowerby) P. Kumm.	☹	☼	–	ND	KY990536	JN974951
*Inocybe godeyi* Gillet	☹	☼	–	ND	FN550897	FN550897
*Inocybe hirtella* Bres.	☹	☼	–	ND	EU523581	EU307822
*Inocybe huijsmanii* Kuyper	☹	☼	–	ND	FR852248	–
*Inocybe ionolepis* Cullington & E. Larss.^c^	○	☼	–	ND	FR852270	–
*Inocybe langei* R. Heim	☹	☼	–	ND	HE687072	JN974962
*Inocybe leptocystis* G.F. Atk.	☹	☼	–	ND	AM882801	AM882801
*Inocybe lilacina* (Peck) Kauffman	☹	☼	–	ND	KY990528	KY990484
*Inocybe mixtilis* (Britzelm.) Sacc.	☹	☼	–	ND	HQ586870	HQ641113
*Inocybe mystica* Stangl & Glowinski	☹	☼	–	ND	NR_158509	–
*Inocybe napipes* J.E. Lange	☹	☼	–	ND	KP308784	KP170955
*Inocybe paludinella* (Peck) Sacc.	☹	☼	–	ND	JF908135	–
*Inocybe praetervisa* Quél.	☹	☼	–	ND	KY033785	KY033785
*Inocybe pusio* P. Karst.	☹	☼	–	ND	FR852266	AY388643
*Inocybe subnudipes* Kühner	☹	☼	–	ND	FN550925	FN550925
*Inocybe tabacina* Furrer-Ziogas	☹	☼	–	ND	HQ586865	HQ641106
*Inocybe terrifera* Kühner	☹	☼	–	ND	–	–
*Inosperma adaequatum* (Britzelm.) Matheny & Esteve-Rav.	☹	☼	–	ND	NR_153149	JQ815407
*Inosperma bongardii* (Weinm.) Matheny & Esteve-Rav.	☹	☼	–	ND	FN550943	FN550943
*Inosperma cookei* (Bres.) Matheny & Esteve-Rav.	☹	☼	–	ND	AM882956	AM882956
*Inosperma erubescens* (A. Blytt) Matheny & Esteve-Rav.	☹	☼	–	ND	AM882951	AM882951
*Inosperma maculatum* (Boud.) Matheny & Esteve-Rav.	☹	☼	–	ND	MH578017	MT228862
*Laccaria amethystina* Cooke	☺	☼	–	W	KU685654	KU685797
*Laccaria bicolor* (Maire) P.D. Orton	☺	☼	–	ND	KM067831	KU685788
*Laccaria laccata* (Scop.) Cooke	☺	☼	–	W	KM067835	KU685859
*Laccaria tortilis* (Bolton) Cooke	☺	☼	–	ND	MG519533	MG519576
*Lacrymaria lacrymabunda* (Bull.) Pat.	☺ X	♠	–	ND	MK968341	MN031155
*Lactarius acris* (Bolton) Gray	X	☼	–	ND	JQ446084	JQ446156
*Lactarius circellatus* Fr.	X	☼	–	ND	FR852038	JN388995
*Lactarius deliciosus* (L.) Gray	☺	☼	–	MW	KJ769672	KF133305
*Lactarius fulvissimus* Romagn.	X	☼	–	ND	FR852027	–
*Lactarius rubrocinctus* Fr.	X	☼	–	ND	UDB005472 (UNITE)	–
*Lactarius scrobiculatus* (Scop.) Fr.	☹ X	☼	–	ND	KX441098	KX441345
*Lactarius serifluus* (DC.) Fr.	☺	☼	–	ND	KT165294	–
*Lactarius subdulcis* (Pers.) Gray	☺ X	☼	–	ND	KX395722	MH872686
*Lactarius tabidus* Fr.	☺	☼	–	ND	KT165309	JN389012
*Lactarius zonarius* (Bull.) Fr.	☺ ☹ (edible in Iran, inedible in Europe)	☼	–	ND	FR852035	MT747331
*Lactifluus glaucescens* (Crossl.) Verbeken	X	☼	–	ND	MT535681	MT554278
*Lactifluus piperatus* (L.) Roussel	☺ ☹ (edible after special preparation)	☼	–	SM	KF220122	KF220215
*Lactifluus vellereus* (Fr.) Kuntze	☺ ☹ X (reported as edible in Turkey by Dogan and Aydin (2013); as inedible in Europe by Heleno et al. (2012))	☼	–	SM	KF220123	KF220216
*Lactifluus volemus* (Fr.) Kuntze	☺	☼	–	W	JQ753936	JQ348387
*Lentinellus cochleatus* (Pers.) P. Karst.	☺	▐	–	ND	AF506417	AF506417
*Lentinellus ursinus* (Fr.) Kühner	☺ (inedible in Europe)	▐	–	ND	MH857168	MH868705
*Lentinellus vulpinus* (Sowerby) Kühner & Maire	X	▐	–	ND	AY513230	–
*Lentinus cyathiformis* (Schaeff.) Bres.	☺ (inedible in Europe)	▐	–	ND	KM411461	KM411477
*Lentinus lepideus* (Fr.) Fr.	☺ ☹ (inedible in Europe)	▐	–	M	KM411454	KM411478
*Lentinus sajor-caju* (Fr.) Fr.	☺	▐	–	MW	OL771751	OL771792
*Lentinus strigosus* Fr.	X	▐	–	ND	KM411451	KM411468
*Lentinus tigrinus* (Bull.) Fr.	☺	▐	–	SM	**ON952481**	MT554282
*Lepiota anthomyces* (Berk. & Broome) Sacc.	○	♠	–	ND	–	–
*Lepiota brunneoincarnata* Chodat & C. Martin	☹	♠	–	ND	MK651615	MK685374
*Lepiota castanea* Quél.	☹	♠	–	ND	MK685380	MK651688
*Lepiota cristata* (Bolton) P. Kumm.	X (considered poisonous in China)	♠	–	ND	LT716026	KY418841
*Lepiota echinella* Quél. & G.E. Bernard	X	♠	–	ND	AY176366	AY176367
*Lepiota felina* (Pers.) P. Karst.	☹	♠	–	ND	MK685381	MK278264
*Lepiota helveola* Bres.	☹	♠	–	ND	MH979466	–
*Lepiota leprica* (Berk. & Broome) Sacc.	○	♠	–	ND	–	–
*Lepiota lilacea* Bres.	☹	♠	–	ND	AY176379	AY176380
*Lepiota metulispora* (Berk. & Broome) Sacc.	○	♠	–	ND	EU681778	MK651673
*Lepiota micropholis* (Berk. & Broome) Sacc.	X	♠	–	ND	–	–
*Lepiota subalba* Kühner ex P.D. Orton	X	♠	–	ND	AY176489	–
*Lepiota subincarnata* J.E. Lange	☹	♠	–	ND	U85329	U85294
*Lepista irina* (Fr.) H.E. Bigelow	☹ ☺ (edible/inedible in the Europe)	♠	–	ND	MH862098	MH873787
*Lepista nuda* (Bull.) Cooke	☺	♠	–	SM	KU215619	DQ071713
*Lepista saeva* (Fr.) P.D. Orton	☺	♠	–	ND	MK785234	MH878430
*Leratiomyces squamosus* (Pers.) Bridge & Spooner	☹ X	♠	–	ND	MH043620	MH036179
*Leucoagaricus americanus* (Peck) Vellinga	☹	♠	–	ND	MT573394	AF482891
*Leucoagaricus badhamii* (Berk. & Broome) Singer	☹	♠	–	ND	GQ329056	–
*Leucoagaricus carneifolius* (Gillet) Wasser	X	♠	–	ND	–	–
*Leucoagaricus holospilotus* (Berk. & Broome) Bon	○	♠	–	ND	–	–
*Leucoagaricus leucothites* (Vittad.) Wasser	☺ ☹ (reported edible in Turkey by Aslim and Ozturk (2011); edible but sometimes caused health problems)	♠	–	SM	MT535726	MT554316
*Leucoagaricus nympharum* (Kalchbr.) Bon	☺	♠	–	ND	EU416310	EU416311
*Leucoagaricus roseoalbus* (Henn.) Heinem.	○	♠	–	ND	–	–
*Leucoagaricus serenus* (Fr.) Bon & Boiffard	☹	♠	–	ND	AY176420	AF482893
*Leucocoprinus birnbaumii* (Corda) Singer	☹	♠	–	ND	MH861267	MH873036
*Leucocoprinus brebissonii* (Godey) Locq.	☹	♠	–	ND	AF482859	AY176446
*Leucocoprinus cepistipes* (Sowerby) Pat.	X ☹	♠	–	ND	LT716023	KY418838
*Leucocoprinus magnusianus* (Henn.) Singer	○	♠	–	ND	–	–
*Leucocybe candicans* (Pers.) Vizzini, P. Alvarado, G. Moreno & Consiglio	☹	♠	–	ND	KJ681027	KJ681051
*Leucocybe houghtonii* (W. Phillips) Halama & Pencakowski	X	♠	–	ND	KY474108	–
*Leucopaxillus compactus* (P. Karst.) Neuhoff	X	♠	–	ND	–	–
*Leucopaxillus giganteus* (Sowerby) Singer	☺ (edible in Europe, considered poisonous in China)	♠	–	M	JQ639151	JQ639152
*Leucopaxillus pinicola* J. Favre	○	♠	–	ND	–	–
*Lyophyllum atratum* (Fr.) Singer	X	♠	–	ND	KJ461896	KJ461895
*Lyophyllum baeospermum* Romagn.	X	♠	–	ND	–	–
*Macrocybe gigantea* (Massee) Pegler & Lodge	☺	♠	–	S	MG867660	AF042591
*Macrolepiota excoriata* (Schaeff.) Wasser	☺ ☹ (edible in Europe)	♠	–	MW	U85313	U85278
*Macrolepiota mastoidea* (Fr.) Singer	☺	♠	–	MW	HM125532	MH867678
*Macrolepiota permixta* (Barla) Pacioni	☺	♠	–	ND	HQ412661	–
*Macrolepiota procera* (Scop.) Singer	☺ (poisonous in China)	♠	–	SM	**ON952483**	AM946456
*Mallocybe dulcamara (Pers.) Vizzini*	☹	☼	–	ND	HQ604787	EU569836
*Mallocybe terrigena* (Fr.) Matheny, Vizzini & Esteve-Rav.	X (edible in China)	☼	–	ND	AM882864	AM882864
*Marasmiellus candidus* (Fr.) Singer	X	▐	–	ND	MT573397	MH867503
*Marasmiellus confluens* (Pers.) J.S. Oliveira	X	♠	–	ND	–	–
*Marasmiellus peronatus* (Bolton) J.S. Oliveira	X (considered edible/poisonous in China)	♠	–	S	AY256706	–
*Marasmiellus ramealis* (Bull.) Singer	X	▐	–	ND	KY404985	KY404980
*Marasmius atrorubens* (Berk.) Mont.	X	♠	–	ND	KP635207	KP635160
*Marasmius corrugatiformis* Singer	X	♠	–	ND	KX148981	–
*Marasmius epiphyllus* (Pers.) Fr.	X	♣	–	ND	JN943599	JN941147
*Marasmius favoloides* Henn.	X	♠	–	ND	–	–
*Marasmius ferrugineus* Berk. & M.A. Curtis	X	♠	–	ND	–	–
*Marasmius haematocephalus* (Mont.) Fr.	X	♠	–	ND	KX148986	EF160083
*Marasmius oreades* (Bolton) Fr.	☺	♠	–	SM	LT716048	KY418864
*Marasmius rotula* (Scop.) Fr.	X	▐	–	ND	JN943598	JN941146
*Marasmius rubroflavus* (Theiss.) Singer	X	♠	–	ND	–	–
*Marasmius wynneae* Berk. & Broome	☺	♠	–	ND	FJ904979	MH868580
*Megacollybia platyphylla* (Pers.) Kotl. & Pouzar	☹ X	▐	–	ND	MT535698	MT554291
*Melanoleuca cognata* (Fr.) Konrad & Maubl.	☺	♠	–	ND	JX429190	JX429180
*Melanoleuca exscissa* (Fr.) Singer	☺	♠	–	S	MT535742	MT554331
*Melanoleuca graminicola* (Velen.) Kühner & Maire	☺	♠	–	ND	JN616438	–
*Melanoleuca grammopodia* (Bull.) Fayod	☺	♠	–	ND	JF908351	MH868277
*Melanoleuca strictipes* (P. Karst.) Jul. Schäff.	☺	♠	–	ND	JX429116	JX429162
*Melanoleuca subpulverulenta* (Pers.) Métrod Note: synonym to *M. friesii* (Bres.) Bon (Antonín et al., 2022) but identification probably tentative.	☺	♠	–	ND	JN616473	–
*Montagnea arenaria* (DC.) Zeller	☺	♠	–	ND	NR_173482	MK278380
*Montagnea haussknechtii* Rabenh.	X	♠	–	ND	–	–
*Mycena acicula* (Schaeff.) P. Kumm.	X	♣ ▐	–	ND	MW540677	MK278389
*Mycena clavicularis* (Fr.) Gillet	X	♣	–	ND	MW540674	AF042637
*Mycena crocata* (Schrad.) P. Kumm.	X	♣	–	ND	JF908492	MH868172
*Mycena filopes* (Bull.) P. Kumm.	X	♣	–	ND	OM473731	–
*Mycena galericulata* (Scop.) Gray	☺	▐	–	ND	DQ404392	MH866154
*Mycena galopus* (Pers.) P. Kumm.	X (edible in China)	♣	Treu and Agerer (1990)	ND	FR846482	AY207250
*Mycena haematopus* (Pers.) P. Kumm.	☹ X	▐	Bermudes et al. (1992)	ND	LT716053	KY418869
*Mycena inclinata* (Fr.) Quél.	X	▐	Desjardin et al. (2008)	ND	MK532829	MK278392
*Mycena metata* (Fr.) P. Kumm.	X	♣	–	ND	MZ315004	–
*Mycena pearsoniana* Dennis ex Singer	☹	♠	–	ND	FN394614	FN394633
*Mycena pelianthina* (Fr.) Quél.	☹	♠	–	ND	FN394549	FN394626
*Mycena polygramma* (Bull.) Gray	X	▐	Treu and Agerer (1990)	ND	MH856239	MH867768
*Mycena pura* (Pers.) P. Kumm.	☹	♠	Treu and Agerer (1990)	ND	KF913023	FN394630
*Mycena rapiolens* J. Favre	X	♣	–	ND	–	–
*Mycena sanguinolenta* (Alb. & Schwein.) P. Kumm.	X	♣	Desjardin et al. (2008)	ND	MH856662	AY207257
*Mycena xantholeuca* Kühner	X	♣	–	ND	MT535719	MT554310
*Mycenastrum corium* (Guers.) Desv.	☺ X (inedible in Europe)	♠	–	S	MH855530	–
*Mycenella salicina* (Velen.) Singer	X	♠	–	ND	JF908497	DQ071720
*Mycetinis alliaceus* (Jacq.) Earle	☺	▐	–	ND	MH856155	MH867679
*Mycetinis scorodonius* (Fr.) A.W. Wilson & Desjardin	☺ X	♣ ▐	–	ND	MH856330	MH867884
*Myxomphalia maura* (Fr.) Hora	X	♠	–	ND	MH856673	MH868189
*Neofavolus suavissimus* (Fr.) J.S. Seelan, Justo & Hibbett	X	▐	–	ND	KM411460	KM411476
*Neolentinus adhaerens* (Alb. & Schwein.) Redhead & Ginns	☺ X	▐	–	ND	HM536096	KJ141188
*Omphaliaster asterosporus* (J.E. Lange) Lamoure	X	♠	–	ND	MZ159333	–
*Omphalina mutila* (Fr.) P.D. Orton	X	♠	–	ND	FJ770399	–
*Omphalina pyxidata* (Bull.) Quél.	X	♠	–	ND	MF319071	MF318927
*Omphalotus olearius* (DC.) Singer	☹	▐	Kotlobay et al. (2018)	SM	AF525061	AF042010
*Ossicaulis lignatilis* (Pers.) Redhead & Ginns	☺	▐	–	ND	DQ825426	AF261397
*Ossicaulis salomii* Siquier & Bellanger	○	▐	–	ND	MT535738	MT554327
*Panaeolus acuminatus* (P. Kumm.) Quél.	☹	♠	–	ND	MH856251	MH867783
*Panaeolus campanulatus* (L.) Quél.	☹	♠	–	ND	JF908522	–
*Panaeolus fimicola* (Fr.) Quél.	☹	♠	–	ND	JF908519	MK278431
*Panaeolus olivaceus* F.H. Møller	☹	♠	–	ND	MH285992	MK278433
*Panaeolus papilionaceus* (Bull.) Quél.	☹	♠	–	ND	MH100681	MK278435
*Panaeolus plantaginiformis* (Lebedeva) E.F. Malysheva	○	♠	–	ND	MK397579	MK397601
*Panaeolus rickenii* Hora	☹	♠	–	ND	JF908523	–
*Panaeolus semiovatus* (Sowerby) S. Lundell & Nannf.	☹ X	♠	–	ND	MH856675	MH868191
*Panaeolus speciosus* P.D. Orton	☹	♠	–	ND	–	–
*Panaeolus teutonicus* Bride & Métrod	☹	♠	–	ND	–	–
*Panellus stipticus* (Bull.) P. Karst.	☹ X	▐	Kotlobay et al. (2018) (chemiluminescence: Shimomura, 1991)	ND	MH855557	MH867062
*Panus conchatus* (Bull.) Fr.	☺	▐	–	M	OL477381	OL477382
*Paragymnopus perforans* (Hoffm.) J.S. Oliveira	X	♣	–	ND	MH856221	AJ406586
*Paralepista flaccida* (Sowerby) Vizzini	☺	♠	–	M	MZ159662	MZ675572
*Parasola auricoma* (Pat.) Redhead, Vilgalys & Hopple	X	♠ ▐	–	ND	MH855972	MH867468
*Parasola hemerobia* (Fr.) Redhead, Vilgalys & Hopple	X	♠ ▐	–	ND	FM163189	FM160720
*Parasola leiocephala* (P.D. Orton) Redhead, Vilgalys & Hopple	X	♠ ▐	–	ND	JN943113	JQ045887
*Parasola miser* (P. Karst.) Redhead, Vilgalys & Hopple	X	♠	–	ND	KY928619	KY928638
*Parasola plicatilis* (Curtis) Redhead, Vilgalys & Hopple	X	♠	–	ND	KY928625	KY928643
*Paraxerula caussei* (Maire) Petersen	☺	▐	–	ND	OL770198	AM946473
*Phaeomarasmius erinaceus* (Fr.) Scherff. ex Romagn.	X	▐	–	M	MH856667	MH868183
*Phaeonematoloma myosotis* (Fr.) Bon	X	♠	–	ND	AF195599	AF195599
*Phellorinia herculeana* (Pers.) Kreisel	X	♠	–	ND	JX984569	–
*Phloeomana speirea* (Fr.) Redhead	X	▐	–	ND	MH856159	MK278448
*Pholiota adiposa* (Batsch) P. Kumm.	☺	▐	–	SM	MT535689	MT554285
*Pholiota astragalina* (Fr.) Singer	X	▐	–	ND	MT187979	MT228845
*Pholiota gummosa* (Lasch) Singer	☺ X	▐	–	ND	MH861987	MH873679
*Pholiota highlandensis* (Peck) A.H. Sm. & Hesler	X	♠	–	ND	MH348872	MH867483
*Pholiota jahnii* Tjall.-Beuk. & Bas	X	▐	–	ND	MT535737	MT554326
*Pholiota populnea* (Pers.) Kuyper & Tjall.-Beuk.	☺ X	▐	–	ND	MG735315	–
*Pholiota scamba* (Fr.) M.M. Moser	X	▐	–	ND	JF908585	–
*Pholiota spumosa* (Fr.) Singer	☺ X	▐	–	ND	MN209776	MN251159
*Pholiota squarrosa* (Oeder) P. Kumm.	☺ (edible but very tough; considered poisonous in China)	▐	–	ND	MN209778	MN251161
*Pholiota squarrosoides* (Peck) Sacc.	X (considered edible/poisonous in China)	▐	–	ND	JF908591	AF261641
*Pholiotina aporos* (Kits van Wav.) Clémençon	X	♠	–	ND	JX968260	JX968376
*Pholiotina arrhenii* (Fr.) Singer	X	♠	–	ND	JX968261	JX968377
*Pholiotina striipes* (Cooke) M.M. Moser	X	♠	–	ND	JX968150	JX968267
*Pholiotina vexans* (P.D. Orton) Bon	X	♠	–	ND	JX968265	JX968380
*Phyllotopsis nidulans* (Pers.) Singer	☺	▐	–	W	MF686492	AF042578
*Pleurotellus chioneus* (Pers.) Kühner	X	▐	–	ND	–	–
*Pleurotus calyptratus* (Lindblad ex Fr.) Sacc.	☺	▐	–	W	EU424283	EU365640
*Pleurotus cornucopiae* (Paulet) Rolland	☺	▐	–	SM	MT535734	MT554324
*Pleurotus djamor* (Rumph. ex Fr.) Boedijn	☺	▐	–	SMW	EU424306	EU365661
*Pleurotus dryinus* (Pers.) P. Kumm.	☺	▐	–	M	EU424292	MH872241
*Pleurotus elongatipes* Peck	○	▐	–	ND	–	–
*Pleurotus eryngii* (DC.) Quél.	☺	♠ ◙	–	SMW	MT535679	MT554276
*Pleurotus fossulatus* (Cooke) Sacc.	☺	▐	–	W	HM998828	U04136
*Pleurotus nebrodensis* (Inzenga) Quél.	☺	♠ ◙	–	SM	HM998835	EU365659
*Pleurotus ostreatus* (Jacq.) P. Kumm.	☺	▐	–	SMW	**ON952482**	MT554286
*Pleurotus pulmonarius* (Fr.) Quél.	☺	▐	–	SMW	LT716061	KY418877
*Pluteus aurantiorugosus* (Trog) Sacc.	X	▐	–	ND	JF908613	AF261579
*Pluteus cervinus* (Schaeff.) P. Kumm.	☺	▐	–	SM	MT535687	MT554283
*Pluteus chrysophaeus* (Schaeff.) Quél.	X	▐	–	ND	MH010881	MH010881
*Pluteus cinereofuscus* J.E. Lange	X	▐	–	ND	MH595963	MK278491
*Pluteus depauperatus* Romagn.	X	▐	–	ND	–	–
*Pluteus exiguus* (Pat.) Sacc.	X	▐	–	ND	FJ774083	–
*Pluteus leoninus* (Schaeff.) P. Kumm.	☺	▐	–	ND	HM562045	HM562234
*Pluteus nanus* (Pers.) P. Kumm.	X	▐	–	ND	MH595974	MK278504
*Pluteus pellitus* (Pers.) P. Kumm.	☺	▐	–	ND	HM562037	HM562225
*Pluteus petasatus* (Fr.) Gillet	☺	▐	–	ND	HM562038	HM562224
*Pluteus punctipes* P.D. Orton	X	▐	–	ND	–	–
*Pluteus romellii* (Britzelm.) Sacc.	☺	▐	–	ND	HM562062	HM562238
*Pluteus salicinus* (Pers.) P. Kumm.	☹ (considered edible in China)	▐	–	ND	HM562051	HM562233
*Pluteus semibulbosus* (Lasch) Gillet	X	▐	–	ND	KR022021	MH867792
*Pluteus thomsonii* (Berk. & Broome) Dennis	X	▐	–	ND	HM562053	HM562230
*Pluteus umbrosus* (Pers.) P. Kumm.	☺	▐	–	ND	HM562140	HM562232
*Pogonoloma macrocephalum* (Schulz.) Sánchez-García	○	♠	–	ND	MH595847	KJ417209
*Psathyrella bivelata* Contu	X	♠	–	S	MT535693	MT554288
*Psathyrella clivensis* (Berk. & Broome) P.D. Orton	X	♠ ▐	–	ND	DQ389683	DQ389683
*Psathyrella fatua* (Fr.) Konrad & Maubl.	X	▐	–	ND	MT535695	MT554290
*Psathyrella hellebosensis* D. Deschuyteneer & A. Melzer	X	♠	–	ND	MT535716	MT554308
*Psathyrella laevissima* (Romagn.) Singer	X	▐	–	ND	–	–
*Psathyrella microrhiza* (Lasch) Konrad & Maubl.	X	♠ ▐	–	ND	MH856265	MH867801
*Psathyrella multipedata* (Peck) A.H. Sm.	X	♠ ▐	–	ND	GQ249282	GQ249291
*Psathyrella obtusata* (Fr.) A.H. Sm.	X	♠ ▐	–	ND	MH860428	–
*Psathyrella pennata* (Fr.) A. Pearson & Dennis	X	♠	–	ND	AM712259	AM712259
*Psathyrella piluliformis* (Bull.) P.D. Orton	☺	▐	–	ND	FN396136	FN396185
*Psathyrella prona* (Fr.) Gillet	X	♠ ▐	–	ND	MH856268	MH867805
*Psathyrella pseudogracilis* (Romagn.) M.M. Moser	X	▐	–	ND	MH856200	MH867728
*Psathyrella spadiceogrisea* (Schaeff.) Maire	X	♠ ▐	–	ND	MK045737	MK045738
*Psathyrella squamosa* (P. Karst.) A.H. Sm.	X	♠ ▐	–	ND	AM712250	AM712250
*Psathyrella tephrophylla* (Romagn.) M.M. Moser	X	♠ ▐	–	ND	AM712270	AM712270
*Pseudoclitocybe cyathiformis* (Bull.) Singer	☺	♠	–	ND	MT535721	MT554311
*Pseudosperma perlatum* (Cooke) Matheny & Esteve-Rav.	☹	☼	–	ND	JQ408767	JQ319698
*Pseudosperma rimosum* (Bull.) Matheny & Esteve-Rav.	☹	☼	–	ND	MF278770	EU600853
*Psilocybe atrobrunnea* (Lasch) Gillet	X	♠	–	ND	HG423575	HG423577
*Psilocybe cyanescens* Wakef.	☹	♠	–	ND	NR_111478	NG_069074
*Psilocybe serbica* M.M. Moser & E. Horak	☹	♠	–	ND	MF958473	MF958467
*Resupinatus applicatus* (Batsch) Gray	X	▐	–	ND	NR_171800	NG_075208
*Rhodocollybia maculata* (Alb. & Schwein.) Singer	X (edible in China)	♠	–	ND	MH857674	MH869212
*Rhodocollybia prolixa* (Fr.) Antonín & Noordel.	X	▐	–	ND	–	MK278563
*Rhodotus palmatus* (Bull.) Maire	X ☹ (maybe poisonous; edibility considered unknown by Heleno et al. (2012))	▐	–	MW	MK287617	MK287618
*Russula acetolens* Rauschert	☺	☼	–	ND	–	–
*Russula alutacea* (Pers.) Fr.	☺ (considered poisonous in China)	☼	–	SM	JF908676	–
*Russula anthracina* Romagn.	☺	☼	(chemiluminescence: Bondar et al., 2012; Gitelson et al., 2012)	S	MW172321	MW182481
*Russula atropurpurea* Peck [non *R. atropurpurea* (Krombh.) Britzelm. (= *R. undulata* Velen.)]	☺	☼	–	ND	JF908691	KU237550
*Russula atrorubens* Quél.	X	☼	–	ND	KX579812	KX812877
*Russula brunneoviolacea* Crawshay	☺	☼	–	ND	AM113956	–
*Russula carminipes* J. Blum	☺	☼	–	ND	–	KU237523
*Russula claroflava* Grove	☺	☼	–	ND	KT933997	KT933858
*Russula cyanoxantha* (Schaeff.) Fr.	☺	☼	(chemiluminescence: Bondar et al., 2012; Gitelson et al., 2012)	S	MW646981	MW646993
*Russula delica* Fr.	☺	☼	(chemiluminescence: Bondar et al., 2012; Gitelson et al., 2012)	W	KX812842	KX812864
*Russula emetica* (Schaeff.) Pers.	☺ ☹ (European *R. emetica* extremely pungent and inedible/toxic; reported by Kaewnarin et al. (2016) as popular edible mushroom in Thailand)	☼	–	M	KX813352	KX812896
*Russula emeticolor* J. Schaeffer	☺	☼	–	S	MT535680	MT554277
*Russula farinipes* Romell	X (edible/poisonous in China)	☼	–	ND	KY800361	KU237561
*Russula foetens* Pers.	☹ X (may cause health problems)	☼	(chemiluminescence: Gitelson et al., 2012)	ND	KT934016	KT933877
*Russula graveolens* Romell	☺	☼	–	ND	KU205298	–
*Russula grisea* Fr.	☺	☼	–	ND	MT738286	MT738262
*Russula heterophylla* (Fr.) Fr.	☺	☼	–	ND	AF418609	AF325309
*Russula integra* (L.) Fr.	☺	☼	–	M	KY582682	KX812899
*Russula ionochlora* Romagn.	☺	☼	–	ND	MW683795	KU237508
*Russula lilacea* Quél.	☺	☼	–	ND	JN944005	JN940592
*Russula luteotacta* Rea	X (edible/poisonous in China)	☼	–	ND	JF908652	KU237512
*Russula nigricans* Fr.	☺	☼	–	S	–	–
*Russula ochroleuca* Pers.	☺	☼	(chemiluminescence: Bondar et al., 2012; Gitelson et al., 2012)	ND	HM189900	KU237519
*Russula ochroleucoides* Kauffman	X	☼	–	ND	–	–
*Russula olivacea* Pers.	☺	☼	–	M	AF418635	KU237492
*Russula pectinata* Fr.	X	☼	–	ND	MW355005	–
*Russula pectinatoides* Peck	☺ X (edible only when very young)	☼	–	ND	EU598185	KU237462
*Russula persicina* Krombh.	X	☼	–	ND	HE687094	KU237494
*Russula perlactea* Murrill	○	☼	–	ND	–	–
*Russula puellaris* Fr.	☺	☼	–	ND	AF418628	KU237515
*Russula queletii* Fr.	☹	☼	–	ND	KT934007	KT933868
*Russula risigallina* (Batsch) Sacc.	☺	☼	–	ND	JF908685	–
*Russula romellii* Maire	☺	☼	–	ND	KT933987	KT933848
*Russula rosea* Pers.	☺	☼	–	S	JN944003	JN940602
*Russula silvestris* (Singer) Reumaux	X	☼	–	ND	KX579800	–
*Russula solaris* Ferd. & Winge	X	☼	–	ND	AF418627	JN940606
*Russula sororia* (Fr.) Romell	X	☼	–	ND	KF318053	–
*Russula torulosa* Bres.	X	☼	–	ND	MZ005531	–
*Russula versicolor* Jul. Schäff.	☺	☼	–	ND	JN944009	JN940594
*Russula veternosa* Fr.	X	☼	–	ND	FR852104	AF325321
*Russula vinosopurpurea* Jul. Schäff.	X	☼	–	ND	FR852115	–
*Russula virescens* (Schaeff.) Fr.	☺	☼	–	SMW	AY061727	AF041548
*Russula xerampelina* (Schaeff.) Fr.	☺	☼	–	W	AY061734	AF218542
*Saproamanita codinae* (Maire) Redhead, Vizzini, Drehmel & Contu	☺	♠	–	ND	–	MK277524
*Sarcomyxa serotina* (Pers.) V. Papp	☺ ○	▐	–	ND	MH856703	MH868220
*Simocybe centunculus* (Fr.) P. Karst.	X	▐	–	ND	MT535746	KT715786
*Sphagnurus paluster* (Peck) Redhead & V. Hofst.	X	♠	–	ND	KP192547	MH873802
*Strobilurus esculentus* (Wulfen) Singer	☺	♠	–	W	MH014049	AY207299
*Strobilurus tenacellus* (Pers.) Singer	X	♠	–	ND	MF063166	MF063102
*Stropharia aeruginosa* (Curtis) Quél.	☺ ☹ X (edible in the Czech Republic)	♠	–	ND	MW492534	MW492637
*Stropharia coronilla* (Bull.) Quél.	☺	♠	–	ND	MH856747	MH868269
*Stropharia melanosperma* (Bull.) Quél.	☺	♠	–	ND	–	–
*Tricholoma acerbum* (Bull.) Quél.	☺ ☹ X (inedible in the Czech Republic)	☼	–	M	MH628231	MK278598
*Tricholoma argyraceum* (Bull.) Gillet	☺	☼	–	ND	GU060278	MK278614
*Tricholoma caligatum* (Viv.) Ricken	☺	☼	–	ND	KU058510	KU058548
*Tricholoma cingulatum* (Almfelt ex Fr.) Jacobasch	☺	☼	–	ND	MH620781	AY207308
*Tricholoma equestre* (L.) P. Kumm.	☺ ☹ (considered poisonous last years, but may include more species)	☼	–	SM	EU186278	AM946471
*Tricholoma fulvum* (Fr.) Bigeard & H. Guill.	☺ ☹ X (inedible in the Czech Republic)	☼	–	ND	KU058514	KU058552
*Tricholoma lascivum* (Fr.) Gillet	☹ (considered edible in China)	☼	–	ND	LT000131	–
*Tricholoma orirubens* Quél.	☺	☼	–	ND	DQ389734	DQ389734
*Tricholoma psammopus* (Kalchbr.) Quél.	X	☼	–	ND	AF377241	–
*Tricholoma robustum* (Alb. & Schwein.) Ricken	☺ X (inedible in southern Europe)	☼	–	ND	AB699669	–
*Tricholoma scalpturatum* (Fr.) Quél.	☺ (considered poisonous in China)	☼	–	ND	JN389305	JN389350
*Tricholoma sulphureum* (Bull.) P. Kumm.	☹	☼	–	M	AY462032	AY462040
*Tricholoma terreum* (Schaeff.) P. Kumm.	☺	☼	–	MW	EU653301	EU653305
*Tricholoma ustale* (Fr.) P. Kumm.	☹ X	☼	–	M	LC574882	AY207306
*Tricholoma ustaloides* Romagn.	X	☼	–	ND	LT000094	–
*Tricholoma vaccinum* (Schaeff.) P. Kumm.	☺ X	☼	–	ND	AF062628	GQ289219
*Tricholomopsis formosa* (Murrill) Singer	X	▐	–	ND	–	–
*Tubaria confragosa* (Fr.) Harmaja	X	▐	–	ND	MF039262	EF051053
*Tubaria conspersa* (Pers.) Fayod	X	♠ ▐	–	ND	MF039274	AF205692
*Tubaria furfuracea* (Pers.) Gillet	☺	♠ ▐	–	W	MZ159504	MH867638
*Tubaria pallidospora* J.E. Lange	X	♠ ▐	–	ND	–	–
*Volvariella iranica* (Fallahyan) Szczepka	○ (probably inedible)	▐	–	ND	–	–
*Volvariella bombycina* (Schaeff.) Singer	☺ X	▐	–	W	MT913623	MK278653
*Volvariella hypopithys* (Fr.) Shaffer	X	♠	–	ND	MN738658	MN738582
*Volvariella murinella* (Quél.) M.M. Moser ex Dennis, P.D. Orton & Hora	X	♠	–	ND	MK412400	MK278657
*Volvariella pusilla* (Pers.) Singer	X	♠	–	ND	HM246494	MK278658
*Volvariella volvacea* (Bull.) Singer	☺	♠	–	M	OM417506	OM373623
*Volvopluteus gloiocephalus* (DC.) Vizzini, Contu & Justo	☹ ☺	♠	–	M	MN738645	MN738593
*Xerula pudens* (Pers.) Singer	☺	▐	–	S	MT535743	MT554333
*Zhuliangomyces ochraceoluteus* (P.D. Orton) Redhead	☺	♠	–	ND	MT863767	MT862275

GenBank accessions in bold were generated in this study. (For species synonyms and distribution data of the species consult Ghobad-Nejhad et al., 2020).

a New to Iran. Voucher: Iran, Tehran, Latmalkan, on soil, IV.2021, Ghobad-Nejhad 4404.

b Information on edibility of the species in China from Wu et al. (2019).

c Occurrence in Iran shown by Crous et al. (2020).

Two concatenated datasets of nLSU + ITS were constructed, one representing the taxa belonging to the order Agaricales (dataset 1), and the other dataset for taxa of Cantharellales, Polyporales, and Russulales (dataset 2). *Contumyces rosellus*, the single Iranian agaric Hymenochaetales, was used as an outgroup for both datasets.

Sequences were aligned using MUSCLE (Madeira et al., 2019). To optimize the alignment, problematic columns were reduced with Noisy 1.5.12 (Dress et al., 2008) and were further identified and removed after careful visual inspection. Special attention was paid to excluding the poorly aligned columns of the ITS region and keeping the finely aligned parts. (Sequences of *Amanita eliae*, *Mycena xantholeuca, Pluteus semibulbosus,* and *Tricholoma ustale* were deleted from the final dataset due to poor alignment.)

### Phylogenetic analyses

The sequence datasets were analyzed using Bayesian inference (BI) executed in MrBayes v. 3.2.7a (Ronquist et al., 2012). MrModeltest 2.3 was implemented to infer the best-fit model of nucleotide evolution for each alignment partition in each dataset (Nylander, 2004). Bayesian analyses were run for 40 (dataset 1) and 20 (dataset 2) million generations for four Markov chain Monte Carlo simulations, in two independent runs at the CIPRES Science Gateway (Miller et al., 2010), with the trees and parameters sampled every 5,000 generations, and the first 25% of the generations were discarded as burn-in. Posterior probabilities (PPs) were calculated from the posterior distribution of the retained trees. Maximum likelihood analyses were executed in raxmlGUI v.1.3 (Silvestro and Michalak, 2010) with the same parameters as used by Ghobad-Nejhad et al. (2021). The Bayesian phylograms were retained for tree visualizations and annotations.

### Edibility, ecological guild and luminescence

The edibility rank of the species (edible, poisonous, inedible) and the ecological guilds (soil saprotrophic, ectomycorrhizal, leaf/litter-inhabiting, wood-inhabiting, parasitic) were assigned based on published literature as well as authors’ knowledge. The edibility of many species is highly subjective and evaluated differently in various countries. Here, the majority of our data are based on central and southern European literature, but even this literature was not necessarily confirmative. Therefore, for some species, more than one rank assignment was inevitably used. Besides the categories “edible” or “poisonous,” category “inedible” was also recognize (noted with symbol X in Table 1) for the species with an unpleasant taste, very small and tiny basidiomata and not usually collected for culinary purposes. Luminescence (bio/chemiluminescent) data were extracted from published literature as mentioned in Table 1 for each species.

### Antioxidant properties

Antioxidant properties of the species were obtained *via* published references as well as own experiments performed in the present study (Tables 1, 2; Supplementary Table 1). A thorough literature survey was performed to extract and summarize the available data on the antioxidant properties of the agaric species. Published references were searched *via* Google Scholar, PubMed, and other standard repositories. Each literature was scrutinized carefully, avoiding poor quality and ambiguous data. Disqualified literature, unpublished data, and papers published in non-standard journals were removed from our analyses. In total, *ca.* 300 literature were surveyed and *ca.* 170 references were cited in this work and in Supplementary material. The majority of studies reported the antioxidant potential as EC_50_ values, i.e., half maximal effective concentration, based on DPPH (2,2-diphenyl-1-picrylhydrazyl) and ABTS (2, 2′-azino-bis-3-ethylbenzothiazoline-6-sulfonic acid) assays. To have an approximate comparison of the antioxidant potential of the species, we tentatively categorized the EC_50_ values as strong, moderate, and weak. For this, the EC_50_ values less than 1 mg/ml were considered as “strong” (S), EC_50_ values ranging from 1 to 10 mg/ml as “moderate” (M), and EC_50_ values more than 10 mg/ml were tentatively considered as “weak” (W) antioxidants (Table 1). For several species, we found different EC_50_ values reported in different studies. We preferred to keep the data as is for any future reference so that we assigned more than one code to classify the antioxidant potential of these species (e.g., SM standing for strong to moderate). (In a number of studies the antioxidant potential had been expressed only as radical scavenging activity% (RSA %). For these, the RSAs >80% were hesitantly considered as strong, RSA 50%–80% as moderate, and RSA < 50% were tentatively considered as weak, paying careful attention also to the values from the antioxidant standards; see Supplementary Table 1). In the cases where the antioxidant potential values were contrasting in different studies, we preferred to keep the data as is for any future reference, and therefore the antioxidant potential of the corresponding species are shown here with the combined codes SM, MW, and SMW, where applicable (Table 1).

**Table 2:** The EC_50_ values and the percentage of radical scavenging activity (RSA) obtained by ABTS assays in this study.

Species	Voucher	RSA % at different concentrations					EC_50_ mg/ml
		0.01 mg/ml	0.025 mg/ml	0.05 mg/ml	0.075 mg/ml	0.1 mg/ml	
*Agaricus arvensis*	Ghobad-Nejhad 4295	14.22	22.07	32.35	43.63	54.92	0.10 ± 0.003
*Agaricus bitorquis*	Ghobad-Nejhad 4284	16.60	22.75	35.67	47.58	59.10	0.08 ± 0.003
*Agaricus iodosmus*	Ghobad-Nejhad 4277	26.85	29.78	37.33	43.88	50.43	0.09 ± 0.021
*Agaricus pseudopratensis*	Ghobad-Nejhad 4278	38.94	39.54	43.20	45.86	48.13	0.11 ± 0.004
*Agrocybe dura*	Ghobad-Nejhad 4299	26.32	27.48	30.41	31.34	33.27	0.31 ± 0.004
*Armillaria mella*	Ghobad-Nejhad 4394	14.25	15.25	16.79	18.53	20.11	0.55 ± 0.016
*Cantharellus alborufescens*	Ghobad-Nejhad 4408	11.45	12.07	12.83	13.79	14.66	1.09 ± 0.06
*Coprinopsis atramentaria*	Ghobad-Nejhad 4406	7.54	9.42	12.15	15.68	18.81	0.34 ± 0.021
*Coprinus comatus*	Ghobad-Nejhad 4407	8.17	10.15	13.45	16.74	19.03	0.34 ± 0.028
*Cortinarius persoonianus*	Ghobad-Nejhad 4206	17.44	19.24	23.25	25.25	28.26	0.27 ± 0.019
*Gymnopilus spectabilis*	Ghobad-Nejhad 4207	18.76	21.13	25.62	29.91	33.20	0.20 ± 0.001
*Hymenopellis radicata*	Ghobad-Nejhad 4204	20.19	20.70	21.50	22.09	22.78	1.05 ± 0.06
*Hypholoma fasciculare*	Ghobad-Nejhad 4201a	29.47	35.57	48.41	60.25	72.09	0.05 ± 0.007
*Lentinus tigrinus*	Ghobad-Nejhad 4397	7.14	8.09	9.68	11.26	12.16	0.75 ± 0.01
*Leucoagaricus leucothites*	Ghobad-Nejhad 4279 4276	19.46	20.80	22.90	25.10	27.50	0.35 ± 0.001
*Melanoleuca exscissa*	Ghobad-Nejhad 4375	28.27	29.61	31.50	34.07	36.90	0.24 ± 0.009
*Pholiota aurivella*	Ghobad-Nejhad 600	38.83	39.48	40.61	41.54	42.84	0.26 ± 0.016
*Pleurotus cornucopiae*	Ghobad-Nejhad 4308	24.75	25.60	27.23	28.77	30.32	0.41 ± 0.001
*Pleurotus eryngii*	Ghobad-Nejhad 1068	32.30	33.14	35.26	37.11	38.40	0.26 ± 0.011
*Pleurotus ostreatus*	Ghobad-Nejhad 4403	6.66	8.20	10.58	12.85	15.33	0.46 ± 0.003
*Pluteus cervinus*	Ghobad-Nejhad 4271	13.14	18.16	27.11	34.96	44.83	0.11 ± 0.002
*Psathyrella bivelata*	Ghobad-Nejhad 4303 4310	36.34	37.11	38.40	39.69	40.18	0.31 ± 0.062
*Russula emeticcolor*	Ghobad-Nejhad 4149	44.45	45.19	46.42	47.66	48.01	0.14 ± 0.028
*Xerula pudens*	Sohrabi 30619	31.78	33.55	35.80	37.55	39.55	0.22 ± 0.002
Trolox							0.023 ± 0.011

Dried basidiomata from 24 species were sampled and examined for their antioxidant potential *via* ABTS assay following Re et al. (1999). Voucher samples were deposited at the Iranian Cryptogamic Herbarium (ICH) herbarium (acronym by Index Herbariorum) or at MG personal collection. The ABTS solution (7 mM) was prepared in 2.45 mM potassium sulfate and was kept at room temperature in the dark for 16 h. The mixture was then mixed with phosphate-buffered saline (PBS) as a control, and the absorbance reached 0.7 ± 0.02 at 734 nm. The extract samples with final concentrations of 0.01, 0.025, 0.05, 0.075, and 0.1 mg/ml were mixed with 980 μl of ABTS solution. The absorbance at 734 nm was measured after 6 min. The percentage of radical scavenging activity was calculated by the following equation, where A stands for absorbance (Öztürk et al., 2011):


$$\begin{array}{l} \mathrm{Scavenging activity}\;\left(\%\right)\\ =\left(A\;\mathrm{control}-A\;\mathrm{sample}\right)/A\;\mathrm{control}\times 100 \end{array}$$

The EC_50_ values were obtained through interpolation from linear regression analysis (Supplementary Figure 1). Trolox was used as a positive control at different concentrations (0.005, 0.01, 0.015, 0.02, 0.025, and 0.03 mg/ml).

## Results

The results of our survey on the resources of agaric species in Iran are summarized in Table 1. Altogether, 558 agaric species from five orders were surveyed for their resources of edible and poisonous species, their ecological guilds, bioluminescence, and antioxidant potential. The two species *Conocybe olivaceopileata* and *Inocybe ionolepis* were added here to the Iranian mycota (see Table 1).

### Phylogeny

**Figure 1:**
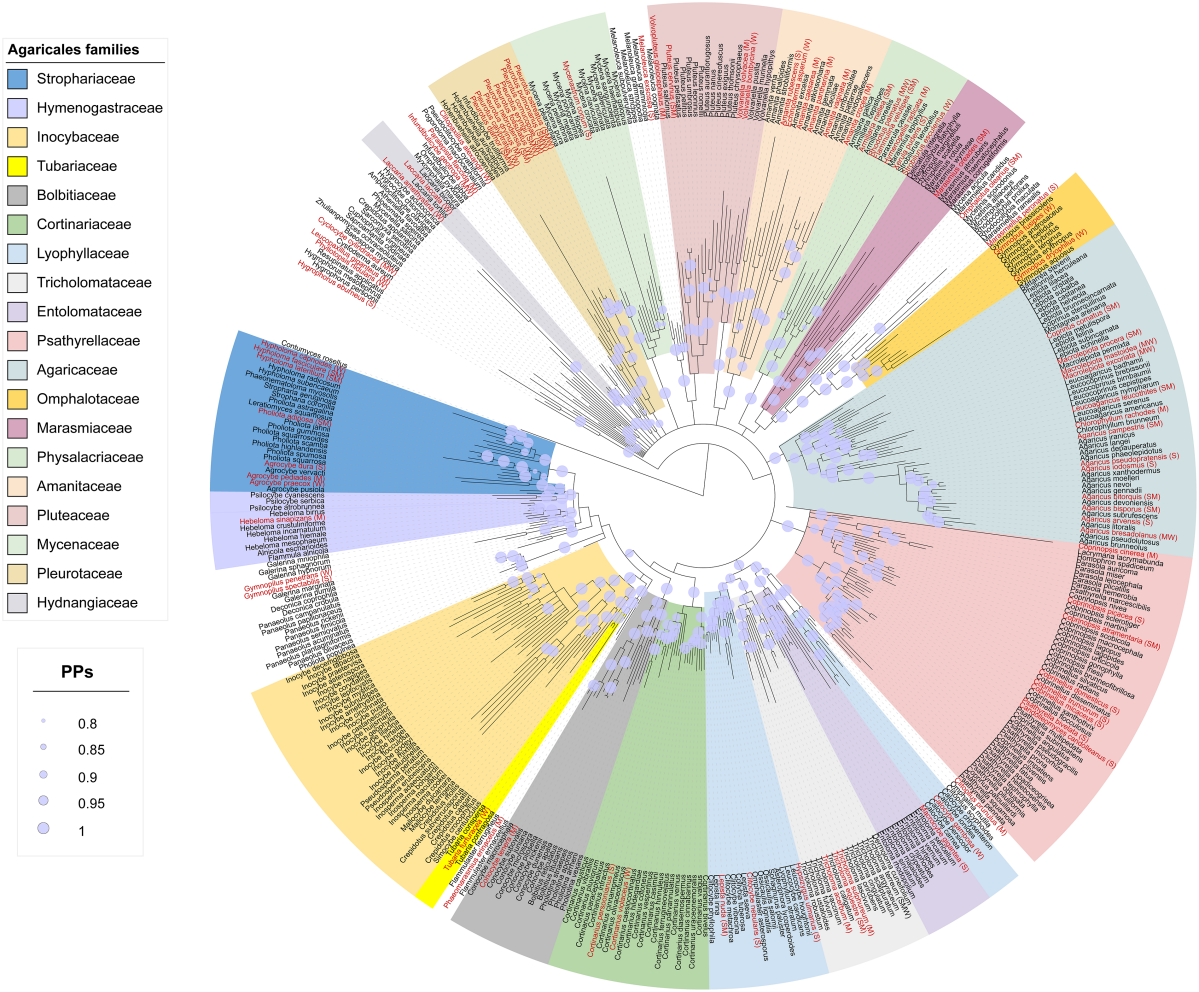
Phylogram from the combined nLSU + ITS sequence dataset representing the phylogenetic relationships of Iranian Agaricales. Posterior probabilities (PPs) ≥ 0.8 are shown as light lilac dots on the nodes. Terminals in red are species with antioxidant activity and the letters inside brackets are the tentative antioxidant codes: S, strong; M, moderate; W, weak (see the text for full details).

The Agaricales dataset consisted of 428 taxa and 1,341 characters of which, 243 characters were constant, 144 variable, and 954 characters were informative. The best-fit evolutionary model suggested by MrModeltest was GTR + I + G for each of the LSU and ITS partitions. The Agaricales phylogram is shown in Figure 1. Nineteen families were phylogenetically retrieved with moderate to good posterior probabilities (PPs) and were shown in colored boxes, while the rest of the taxa were incertae sedis or received low to moderate branch support.

The species with antioxidant data were distributed in all the families shown in colored boxes except for the two families Entolomataceae and Inocybaceae (Figure 1). For some families such as Bolbitiaceae, Marasmiaceae, and Tubariaceae, there was only a single species with antioxidant activity, while other families such as Agaricaceae, Psathyrellaceae, and Pleurotaceae contained several antioxidant species.

**Figure 2:**
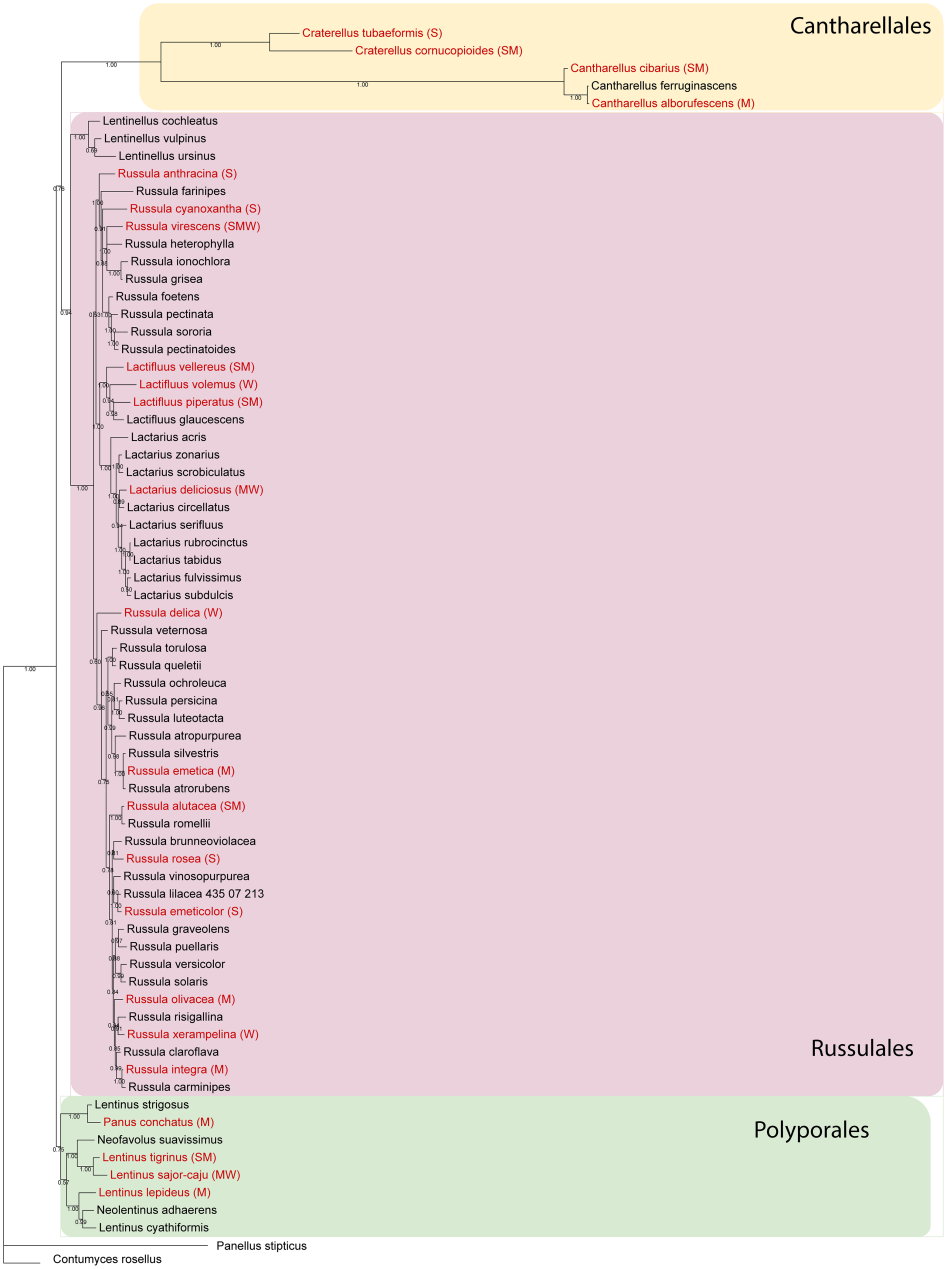
Phylogram from the combined nLSU + ITS sequence dataset representing phylogenetic relationships of the Iranian Cantharellaeles, Russulales, and Polyporales. Posterior probabilities are shown below branches. Terminals in red are species with antioxidant activity and the letters inside brackets are the tentative antioxidant codes: S, strong; M, moderate; W, weak (see the text for full details).

Dataset 2 (Cantharellales, Polyporales, Russulales) consisted of 71 taxa and 1,528 characters of which, 279 characters were constant, 347 variable, and 902 characters were informative. The best-fit evolutionary model as suggested by MrModeltest was GTR + G for each of the LSU and ITS partitions. The phylogram obtained from the analyses of dataset 2 is presented in Figure 2. The orders Polyporales, Russulales, and Cantharellales were retrieved as moderate to well-supported monophyletic clades (PPs 0.75, 0.94, and 1.00, respectively). (For Polyporales, the two families Panaceae and Polyporaceae were not retrieved. Moreover, *Panellus stipticus* found a position close to the outgroup *Contumyces rosellus*, and we could not solve this.) The species with antioxidant data were distributed within the three orders in the phylogram (Figure 2). Out of six Cantharellales agaric members in Iran (Ghobad-Nejhad et al., 2020), four species have antioxidant data (Figure 2; *Craterellus cinereus* had no good LSU/ITS, so is missing in the phylogeny here). Polyporales has nine agaric species in Iran, four of which possess antioxidant activity (Figure 2). Russulales has 60 agaric species in Iran (Ghobad-Nejhad et al., 2020) from which, 16 species have antioxidant data (Figure 2; Table 1). *Contumyces rosellus* is the only Hymenochaetales agaric in Iran and has no antioxidant data.

Altogether, there were 50 agaric species lacking both ITS and LSU sequences and so did not appear in the phylogenetic analyses (Table 1); these species also lacked antioxidant data, except for *Russula nigricans* which was scored as a “strong” antioxidant species (Table 1).

### Edibility, ecological guild, and luminescence

**Figure 3:**
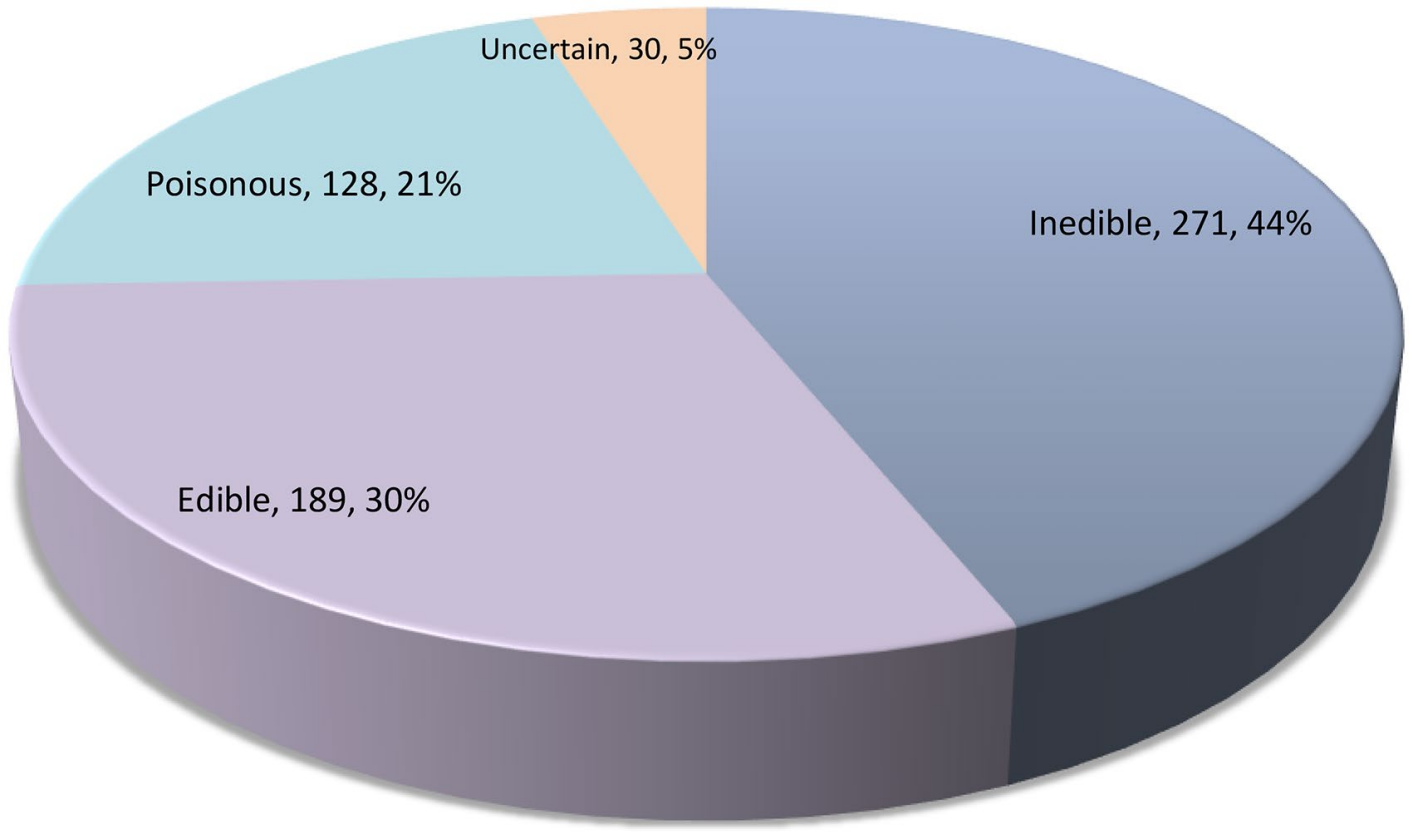
The number of Iranian agaric species in each edibility category.

**Figure 4:**
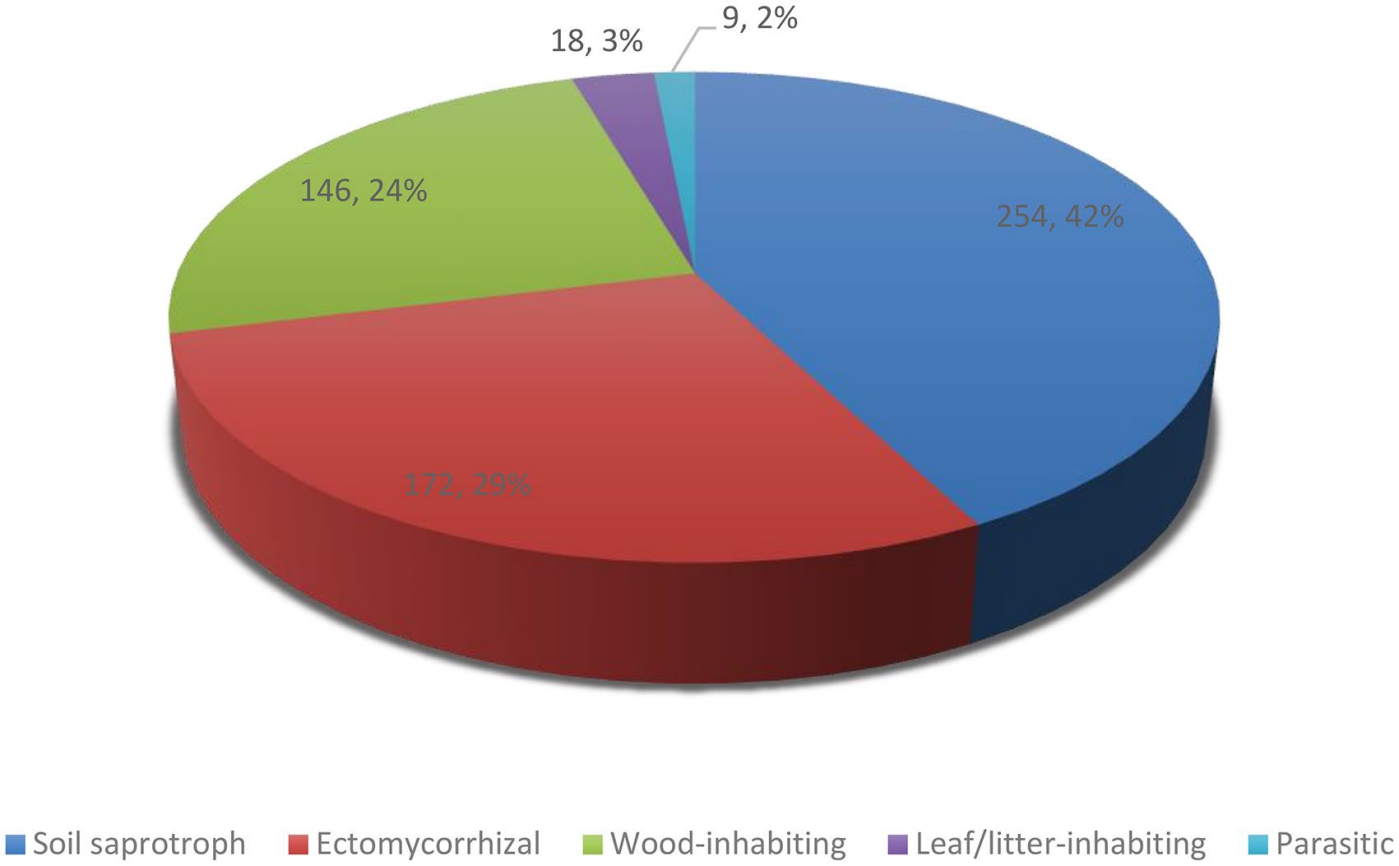
The number of Iranian agaric species in each ecological guild.

Results of the survey on edibility, ecological guilds, and luminescence of agaric species occurring in Iran are shown in Table 1 and Figures 3, 4. It is revealed that about 189 species of agarics in Iran can be classified as edible, 128 species as poisonous, and 271 species as inedible (Table 1; Figure 3). Moreover, 10 species can be assigned as edible only if well-cooked, whereas the edibility of 30 species is uncertain or unknown.

Concerning ecological guilds, our results show that about 254 species of agarics in Iran are soil saprotrophic, 172 species ectomycorrhizal, 146 species wood-inhabiting, 18 species leaf/litter-inhabiting, and nine species are parasitic (Table 1; Figure 4). Parasitic species include *Armillaria borealis*, *A. cepistipes, A. gallica*, *A. mellea*, *Collybia tuberosa, Pleurotus eryngii, P. nebrodensis* which are sapro-parasitic, *Gymnopus fusipes* which is wood-inhabiting parasitic, and *Asterophora lycoperdoides* which grows on basidiomata of *Lactarius* and *Russula* species (Table 1).

Among 558 agaric species in Iran, 19 species are categorized as luminescent (Table 1). These include *Armillaria* (four spp.), *Collybia tuberosa*, *Flammulina velutipes*, *Mycena* (six spp.), *Omphalotus olearius*, *Panellus stipticus*, and *Russula* (five spp.). The six species with chemiluminescence include *Russula anthracina, R. cyanoxantha, R. delica, R. foetens, R. ochroleuca*, as well as *Panellus stipticus.*

### Antioxidant potential

**Figure 5:**
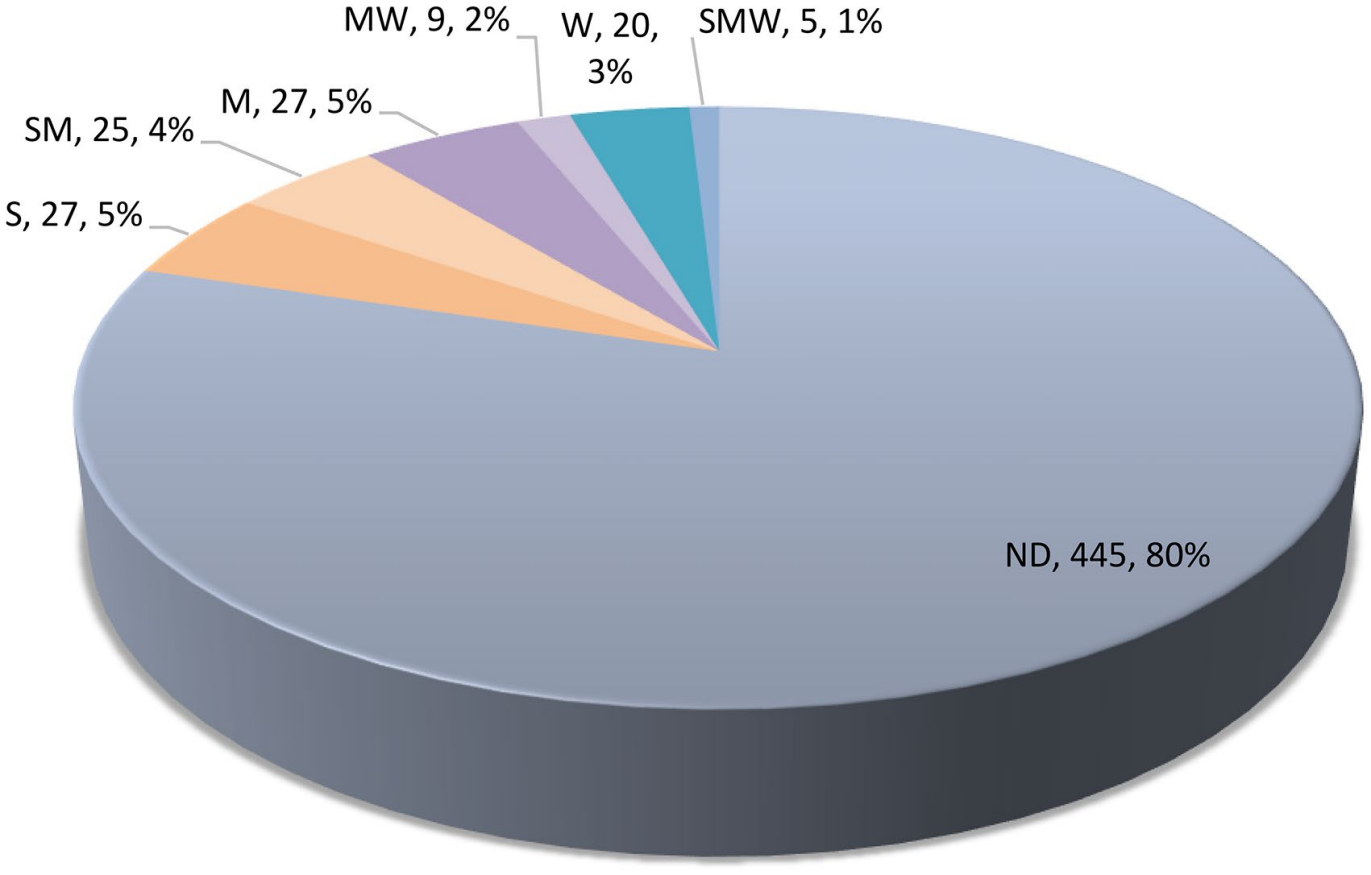
Categorization of antioxidant potential of Iranian agaric species. S, strong; M, moderate; W, weak; ND, not determined. See the text for full details.

Results of our survey on the antioxidant potential of agaric species occurring in Iran are shown in Table 1 and Figure 5 (see also Supplementary Table 1 for details on the antioxidant potential of the species and the corresponding references). According to the results, antioxidant activity data is available for 113 species phylogenetically distributed in four orders (Agaricales, Cantharellales, Russulales, Polyporales) and 21 agaric families including 17 families in the Agaricales (Strophariaceae, Hymenogastraceae, Tubariaceae, Bolbitiaceae, Cortinariaceae, Lyophyllaceae, Tricholmataceae, Psathyrellaceae, Agaricaceae, Omphalotaceae, Marasmiaceae, Physalacriaceae, Amanitaceae, Pluteaceae, Mycenaceae, Pleurotaceae, Hydnangiaceae; Figure 1), as well as Hydnaceae (=Cantharellaceae), Russulaceae, Polyporaceae, and Panaceae (Figure 2, families not shown on the tree). However, 445 species still lack information on their antioxidant potential (for a handful of species, the available antioxidant values in the literature had been expressed only by other methods such as TEAC and FRAP; as far as these cases were very few, they were not taken into account here, to keep the rest of the data comparable). The antioxidant potential of 24 species was assayed in this study and their EC_50_ values are reported in Table 2. Species assayed for the first time in this study included: *Agaricus iodosmus, A. pseudopratensis, Agrocybe dura, Cantharellus alborufescens, Cortinarius persoonianus, Hymenopellis radicata, Melanoleuca exscissa, Psathyrella bivelata, Russula emeticolor,* and *Xerula pudens* (Table 2; Supplementary Table 1). In general, the EC_50_ values of the agaric species ranged between 0.0015 mg/ml (for *Psathyrella candolleana*) up to 31.42 mg/ml (for *Tricholoma terreum*).

Among the 113 species having antioxidant data, 27 species could roughly be classified as “strong,” 25 species as “strong to moderate,” 27 species as “moderate,” nine species as “moderate to weak,” and 20 species could be tentatively regarded as “weak” antioxidants (Figure 5). Some of the species in the S category are *Agaricus arvensis*, *Agrocybe dura*, *Amanita rubescens*, *Candolleomyces candolleanus*, *Clitocybe nebularis*, *Coprinellus micaceus, Coprinopsis picacea, Craterellus tubaeformis, Gymnopilus spectabilis, Hygrophorus eburneus, Hypsizygus ulmarius, Macrocybe gigantean, Marasmiellus peronatus, Mycenastrum corium, Russula anthracina, Russula cyanoxantha, Russula emeticolor, Russula nigricans, Russula rosea,* and *Xerula pudens* (Table 1).

The overall phylogenetic distribution of the agaric species with antioxidant data is shown in Figures 1, 2. *Russula nigricans* was the only species in our dataset with antioxidant data but lacked LSU/ITS DNA sequences in GenBank, so could not be used in our phylogenetic analyses.

## Discussion

In this study, we comprehensively investigated the resources of agarics in Iran. Indeed, no published data have yet been available on number of recorded edible, poisonous, and other agarics in Iran, so the present work fills in these gaps. It is shown that there are currently about 189 edible, 128 poisonous, 254 soil saprotrophic, 172 ectomycorrhizal, 146 wood-inhabiting, 18 leaf/litter-inhabiting, 9 parasitic, and 19 luminescent agaric species in the country. The two species *Conocybe olivaceopileata* and *Inocybe ionolepis* were newly added to the Iranian mycota, new DNA sequences were obtained from Iranian samples, and the first phylogenetic reconstruction was provided for agarics of Iran. Evidently, this work is not final and therefore further studies of Iranian fungal diversity would add new species to the list presented here. About 500 agaric species belonging to the five orders Agaricales, Cantharellales, Polyporales, Russulales, and Hymenochaetales were phylogenetically analyzed based on nLSU + ITS sequence datasets. Thorough analyses with additional gene regions and vouchered samples must be utilized in the future to resolve the phylogenetic relationships of Iranian agarics. Yet, the preliminary phylogenetic analysis of agaric species presented here would help to inspire the investigation of many taxa in need of taxonomic revision. Phylogeny backbones can be used for visualization of the phylogenetic distribution of species possessing particular characteristics, herein, antioxidant potential, but also other features in the future. For instance, phylogenetic assessments have been used to screen the pleuromutilin-producing basidiomycete species (Hartley et al., 2009), fungal strains capable of degrading industrial compounds (Navarro et al., 2021), or other natural products (Adamek et al., 2019).

For a few species, the edibility assignment was based on own observation in Iran, but as stated earlier, most of the species were categorized based on available knowledge on central and southern European species. (It might be relevant to note that a number of previous studies have shown a high similarity of the Iranian mycota to that of Europe, e.g., Ghobad-Nejhad et al., 2012; Ghobad-Nejhad and Bernicchia, 2019.) Basically, edibility assignments should always be regarded with caution and it is generally recommended to avoid consuming raw or insufficiently identified mushrooms. There are still noticeable gaps in the knowledge of edible/poisonous mushrooms identification in Iran and the level of education, public awareness, and citizen science is far from medium standards. Concerning usage of edible fungi in Iran, published references are lacking, and our available data is fragmentary. In the reports and statistics on mushroom poisoning in Iran, there is no proper documentation of the species involved or at best, the species are only ambiguously characterized (Kiarsi et al., 2019).

The present work calculated as many as about 172 ectomycorrhizal agaric species for Iran. Ectomycorrhizal fungi are essential components of forest ecosystems to supply the symbiont trees with water and nutrients such as phosphorus and nitrogen, and therefore are highly important in forest sustainability (Varma and Hock, 2013). A large number of ectomycorrhizal agarics are also edible and may be harvested in the wild for culinary use, so they are in need of immediate conservation actions (Vaario and Matsushita, 2021); this is the case, especially with the *Cantharellus* species in northern Iran (Parad et al., 2018, 2020).

In this study, we listed 146 wood-inhabiting agaric species for Iran. There have been several studies on the diversity and taxonomy of wood-inhabiting aphyllophoroid fungi in Iran (e.g., Hallenberg, 1981; Ghobad-Nejhad and Hallenberg 2012; Amoopour et al., 2016; Ghobad-Nejhad and Langer, 2017; Nazari Mahroo et al., 2018; Ghobad-Nejhad and Bernicchia, 2019) but agarics growing on wood in Iran have not been studied systematically. Wood rotting fungi play a key role in terrestrial carbon cycling and have high potential in biotechnology, enzyme industry, biorefinery, and bioremediation of waste material and recalcitrant compounds (Gadd, 2001; Nguyen et al., 2018; Mäkelä et al., 2021). While Polyporales members are best known for their wood decomposition ability, genomic studies have revealed that several Agaricales taxa have evolved the enzymatic machinery comparable to the white-rot Polyporales (Floudas et al., 2020; Ruiz-Dueñas et al., 2020).

Another aspect surveyed in this study for the Iranian agaric species was bioluminescence. Bioluminescence, i.e., the ability of organisms to emit visible light, has been developed independently in the evolution of different organisms. Concerning fungi, 109 fungal taxa are known to exhibit bioluminescence all of which (except one Xylariales) are white-spored saprotrophic Basidiomycota distinguished in four phylogenetic lineages (Chew et al., 2015; Ke and Tsai, 2022) all sharing the same type of luciferin and luciferase (Oliveira et al., 2012). Interestingly, it has been shown that luminescence could be linked to the antioxidant/radical scavenging defense mechanism against some environmental stress factors (Vydryakova and Bissett, 2016; Oba et al., 2017). Moreover, the fungal bioluminescence capacity can be used in environmental biomonitoring of metals or organic compounds and to develop toxicity tests (Ke and Tsai, 2022).

In this work, a thorough survey was done to reveal the antioxidant potential of 558 agaric species and a new approach was used to combine antioxidant data with phylogeny of the species. Ten species were subjected to antioxidant analyses for the first time, belonging to the genera *Agaricus, Agrocybe, Cantharellus, Cortinarius, Hymenopellis, Melanoleuca, Psathyrella, Russula,* and *Xerula.* ABTS assay is one of the most frequently used method for quantification of antioxidant activity of mushrooms. Numerous antioxidant assays have been introduced which are usually classified into two groups based on the mechanism of action: single electron transfer and hydrogen atom transfer (Tan and Lim, 2015; Xiao et al., 2020). Compared to other methods, ABTS has the advantage of involving more or less both mechanisms (Prior et al., 2005). Yet, more examinations are required to fully investigate the antioxidant capacity of the species studied here, and to quantify and characterize the underlying bioactive compounds. Here, we could resume antioxidant data for 20% of agaric species (113 spp.), but noted that 80% of the species (445 spp.) have no antioxidant data. This is noteworthy compared to the fact that antioxidant tests are among the most popular bioactivity assays and it may show that macrofungi have remained little studied in this regard. The highest antioxidant capacities (the lowest EC_50_ values) were shown by the species categorized as S (27 spp.) and then as SM (25 spp.; Table 1; Figure 5). As noted earliers, in several cases, various EC_50_ values had been reported in different studies for some species, so that we assigned more than one code for them. We emphasize that such classification is approximate and for detailed comparisons, more precise methods are recommended to be applied. For five species, the EC_50_ measures ranged significantly, in a way that the code assignment could only be expressed as SMW: *Pleurotus djamor, P. eryngii, P. ostreatus, P. pulmonarius,* and *Russula virescens.* Of course, differences in the solvents, standards, modifications in the assays procedures, and even identification issues can account for the different measures under the same species name. Ideally, the identity of the voucher specimens should be fully characterized and the species should be assayed with exactly the same procedure so as to be able to have the best quality comparisons. In general, for the studies where both ABTS and DPPH assays had been conducted, ABTS values seemed to slightly outperform the DPPH values, showing lower EC_50_ measures. Many of the species in the S or “strong” antioxidant category are edible: *Agaricus arvensis*, *Agrocybe dura*, *Amanita rubescens*, *Candolleomyces candolleanus*, *Clitocybe nebularis*, *Craterellus tubaeformis, Hygrophorus eburneus, Hypsizygus ulmarius, Macrocybe gigantea, Russula anthracina, R. cyanoxantha, R. emeticolor, R. nigricans, R. rosea,* and *Xerula pudens* (Table 1). Oxidative stress is the root of a cascade of numerous acute and chronic human diseases (Kosanic et al., 2013). Diets rich in natural antioxidants enforce the native defense system and protect against oxidative damage (Ferreira et al., 2009). Mushroom species that are edible and possess high level of biological activities with perspectives on promoting human health are considered noteworthy candidates for developing functional foods and nutra-pharmaceutical products (Kozarski et al., 2015; Lu et al., 2020; Niego et al., 2021; Shaffique et al., 2021; El Sheikha, 2022). It is evident that thorough analyses are needed to fully characterize the mycochemical constitutes of such species and their various bioactivities.

Our results pave the avenue for advanced studies on edible, poisonous, saprotrophic, ectomycorrhizal, wood-inhabiting, parasitic, luminescent, and antioxidant species of agarics of Iran. Twenty percent of the Iranian agaric species possess antioxidant activity, phylogenetically distributed in four orders and 21 agaric families. About 5% of the antioxidant species can be considered strong antioxidants, many of which are also edible and could be utilized for the development of functional foods. Various edible agaric species are grown commercially in the world, while only 1–2 are commonly grown in Iran (personal comm.). Ectomycorrhizal and wood-inhabiting species are important components of forest sustainability. Forests in Iran are very scanty, comprising less than 10% of the total country area, and are on the verge of severe depletion due to numerous anthropological and environmental threats. Yet, Iranian old-growth forests, categorized as part of the northern hemisphere glacial refugia (Ghobad-Nejhad et al., 2012, 2020), harbor a rich reservoir of agaric fungi with diverse characteristics and beneficial aspects. Resources of Iranian agarics provide valuable opportunities for biotechnology and mycochemistry, and should be regarded for preservation and habitat conservation. Our preliminary phylogenetic trees would guide the selection of agaric taxa to be examined in the future for taxonomic revisions, biotechnological applications, and applied phylogeny studies. The thorough survey of antioxidant data of 558 agaric species would provide the state of the knowledge on agarics examined so far and the remaining gaps to be filled in the future.

## Data availability statement

The datasets presented in this study can be found in online repositories. The names of the repository/repositories and accession number(s) can be found in the article/Supplementary material.

## Author contributions

MG-N conceptualized and designed the study, performed the molecular study and provided the first draft. VA contributed to the trait assignments. MG-N, VA, and EL wrote the manuscript. MG-N and MM performed the experiments. All authors contributed to the article and approved the submitted version.

## Funding

This work was supported by the Center for International Scientific Studies & Collaboration (CISSC), Ministry of Science, Research, and Technology of Iran. The studies of VA were enabled by the support provided to the Moravian Museum by the Ministry of Culture of the Czech Republic as part of its long-term conceptual development program for research institutions (DKRVO, ref. MK000094862).

## Conflict of interest

The authors declare that the research was conducted in the absence of any commercial or financial relationships that could be construed as a potential conflict of interest.

## Publisher’s note

All claims expressed in this article are solely those of the authors and do not necessarily represent those of their affiliated organizations, or those of the publisher, the editors and the reviewers. Any product that may be evaluated in this article, or claim that may be made by its manufacturer, is not guaranteed or endorsed by the publisher.

## Footnotes

^1^ www.indexfungorum.org

^2^ www.mycobank.org
