# Supplementary material for: Resources of Iranian agarics (Basidiomycota) with an outlook on their antioxidant potential
Source: Front Microbiol. 2022 Oct 28;13:1015440. doi: 10.3389/fmicb.2022.1015440 (PMC9650231; doi:10.3389/fmicb.2022.1015440)
Supplement: Supplementary file 1 [file Data_Sheet_1.docx]

**Supplementary Materials.**

**Supplementary Table 1** Details on antioxidant activity of agaric species occurring in Iran and the pertaining literature.

| **Species** | **Antioxidant potential** |
| --- | --- |
|  |  |
| *Agaricus arvensis* Schaeff. | EC_50_ 0.8 mg/ml, 90.7 % (DPPH) (1)  EC_50_ 15.85 mg/ml (DPPH) (2)  IC_50_ 0.1 mg/ml (this study) |
| *Agaricus bisporus* (J.E. Lange) Imbach | EC_50_ 1.4 – 3.2 mg/ml, scavenging activity 62.7 – 89.3 % (DPPH) (1)  IC_50_ 0.99 – 2.65 mg/ml (DPPH), 0.24 – 1.13 mg/ml (ABTS) (3)  EC_50_ 9.61 mg/ml (DPPH) (2)  IC_50_ ≥ 0.4 mg/ml (ABTS) and ≥ 0.8 (DPPH) (4)  EC_50_ 3.82 mg/ml (DPPH) (5) |
| *Agaricus bitorquis* (Quél.) Sacc. | EC_50_ 1.2 mg/ml, 91.3 % (DPPH) (1)  scavenging activity 0.39 – 3.93 mg/ml (DPPH), 0.09 – 1.63 mg/ml (ABTS) (3)  EC_50_ 3.44 mg/ml (DPPH) (5)  IC_50_ 0.08 mg/ml (this study) |
| *Agaricus bresadolanus* Bohus | scavenging activity 12.28 – 24.70 % (DPPH), 64.11 – 75.98 % (ABTS) (6) |
| *Agaricus campestris* L. | EC_50_ 1.6 mg/ml, 84.3 % (DPPH) (1)  IC_50_ 0.416 mg/ml (DPPH) (7)  EC_50_ 1.18 mg/ml (DPPH) (5) |
| *Agaricus iodosmus* Heinem. | IC_50_ 0.09 mg/ml (this study) |
| *Agaricus pseudopratensis* (Bohus) Wasser | IC_50_ 0.11 mg/ml (this study) |
| *Agaricus subrufescens* Peck | EC_50_ 3.0 – 3.2 mg/ml (DPPH) (8)  EC_50_ 2.15 mg/ml (DPPH) (9)  EC_50_ 5.02 mg/ml (DPPH) and 6.77 mg/ml (ABTS)(10) |
| *Agrocybe dura* (Bolton) Singer | IC_50_ 0.16 mg/ml (this study) |
| *Agrocybe pediades* (Fr.) Fayo | scavenging activity 65.5% (DPPH) (standard vit. C 97.2%) (11) |
| *Agrocybe praecox* (Pers.) Fayod | IC_50_ 12.5 mg/ml (ABTS) (12) |
| *Amanita caesarea* (Scop.) Pers. | EC_50_ 7.41 mg/ml (DPPH) (13) |
| *Amanita crocea* (Quél.) Singer | EC_50_ 7.94 mg/ml (DPPH) (14)  IC_50_ 1.69 mg/ml (DPPH) and 0.92 (ABTS) (a polysaccharide) (15) |
| *Amanita pantherina* (DC.) Krombh. | EC_50_ 3.89 mg/ml (DPPH) (13) |
| *Amanita rubescens* Pers. | IC_50_ 0.114 – 0.186 mg/ml (DPPH) (16) |
| *Amanita vaginata* (Bull.) Lam. s.l. | EC_50_ 1.8 mg/ml (DPPH) (17) |
| *Armillaria mellea* (Vahl) P. Kumm. | EC_50_ 2.96 – 8.62 mg/ml (DPPH) (18)  IC_50_ 1.06 – 1.32 mg/ml (DPPH) (19)  IC_50_ 6.44 mg/ml (DPPH) (20)  IC_50_ 1.20 mg/ml (DPPH) (21)  IC_50_ 0.55 mg/ml (this study) |
| *Calocybe gambosa* (Fr.) Donk | IC_50_ 7.08 – 34.60 mg/ml (DPPH) (22) |
| *Cantharellus alborufescens* (Malençon) Papetti & S. Alberti | IC_50_ 1.12 mg/ml (this study) |
| *Cantharellus cibarius* Fr. | IC_50_ 0.158 – 0.192 mg/ml (DPPH) (16)  IC_50_ 0.264 – 0.548 mg/ml (DPPH) (IC_50_ Fe chelating ability 0.016 –0.579 mg/ml, H2O2 scavenging activity 0.00064 – 0.0012 mg/ml) (specied det. unconfirmed) (23)  EC_50_ 19.65 mg/ml (DPPH) (**Error! Reference source not found.**)  EC_50_ 7.41 mg/ml (DPPH?) (24)  scavenging activity 61.7 –70.5% (DPPH) (standard 93.8%) (25) |
| *Chlorophyllum rhacodes* (Vittad.) Vellinga | EC_50_ 5.32 mg/ml (DPPH) (26)  EC_50_ 3.4 mg/ml (DPPH) (27) |
| *Clitocybe nebularis* (Batsch) P. Kumm. | IC_50_ 0.046 mg/ml (DPPH) (standard vit. C 0.006) (28) |
| *Clitopaxillus alexandri* (Gillet) G. Moreno, Vizzini, Consiglio & P. Alvarado | EC_50_ 28.72 (mg/ml) (DPPH) (29) |
| *Clitopilus prunulus* (Scop.) P. Kumm. | EC_50_ 1.75 mg/ml (DPPH) (30) |
| *Conocybe tenera* (Schaeff.) Fayod | EC_50_ 5.13 mg/ml (DPPH) 3.21 mg/ml (ABTS) (31) |
| *Coprinellus domesticus* (Bolton) Vilgalys, Hopple & Jacq. Johnson | IC_50_ 0.250 – 0.397 mg/ml (DPPH) (32) |
| *Coprinellus micaceus* (Bull.) Vilgalys, Hopple & Jacq. Johnson | IC_50_ 0.5 mg/ml (DPPH) (33)  scavenging activity 81.45% (DPPH) (34) |
| *Coprinellus truncorum* (Scop.) Redhead, Vilgalys & Moncalvo | IC_50_ 0.065 mg/ml (DPPH) (35) |
| *Coprinopsis atramentaria* (Bull.) Redhead, Vilgalys & Moncalvo | EC_50_ 2.48 – 3.87 mg/ml (DPPH) (36)  IC_50_ 0.34 mg/ml (this study) |
| *Coprinopsis cinerea* (Schaeff.) Redhead, Vilgalys & Moncalvo | scavenging activity 50% (DPPH) (37)  EC_50_ 4.24 mg/ml (DPPH) (38) |
| *Coprinopsis picacea* (Bull.) Redhead, Vilgalys & Moncalvo | IC50 0.45 mg/ml (DPPH) (39) |
| *Coprinus comatus* (O.F. Müll.) Pers. | EC_50_ 0.86 – 8.9810 mg/ml (DPPH) (40)  scavenging activity 84.5% (DPPH) (standard BHA 93.6%) (41)  IC_50_ 0.024 mg/ml (DPPH) (35)  IC_50_ 0.32 mg/ml (this study) |
| *Cortinarius persoonianus* Bidaud | IC_50_ 0.28 mg/ml (this study) |
| *Cortinarius violaceus* (L.) Gray | EC_50_ 15.70 mg/ml (DPPH) (13) |
| *Craterellus cornucopioides* (L.) Pers. | IC_50_ 0.019 mg/ml (DPPH) (standard vit. C 0.006 mg/ml) (42)  IC_50_ 5.26–12.01 mg/ml (DPPH) (43) |
| *Craterellus tubaeformis* (Fr.) Quél. | EC_50_ 0.6 mg/ml (DPPH) and 0.27 mg/ml (ABTS) (44) |
| *Cyclocybe cylindracea* (DC.) Vizzini & Angelini | EC_50_ 7 – 21.5 mg/ml (DPPH) (45)  scavenging activity 40% (DPPH) (standard catechin 65.22%) (46) |
| *Echinoderma asperum* (Pers.) Bon | DPPH ‘negative’ (47) |
| *Flammulina velutipes* (Curtis) Singer | IC_50_ > 50 mg/ml (DPPH) (48)  scavenging activity 57.93%–66.75% (DPPH) (polysaccharides) (standard BHA 99.82%) (49)  scavenging activity 67.37% (polysaccharides) (DPPH) and 67.87% (H2O2) (50)  IC_50_ 0.97 mg/ml (DPPH) (51)  IC_50_ 0.2 – 0.57 mg/ml (DPPH) (52)  EC_50_ 0.46 ­– 0.63 mg/ml (DPPH) (53)  EC_50_ 1.6±0.4 mg/ml (DPPH) (**Error! Reference source not found.**) |
| *Gymnopilus penetrans* (Fr.) Murrill | IC_50_ 17.49 ± 0.21 mg of dry extract per mg DPPH (54) |
| *Gymnopilus spectabilis* (Weinm.) A.H. Sm. | scavenging activity ≤ 80.33% (DPPH) (standard BHA ≤ 93.33) (55)  IC_50_ 0.19 mg/ml (this study) |
| *Gymnopus dryophilus* (Bull.) Murrill | scavenging activity 7.41 – 35.10 % (DPPH), 20.58 – 59.11 % (ABTS) (6) |
| *Gymnopus fusipes* (Bull.) Gray | EC_50_ 15.46 ± 0.39 mg/ml DPPH (56) |
| *Hebeloma sinapizans* (Paulet) Gillet | EC_50_ 6.96 mg/ml (DPPH) (standard Trolox 0.04 mg/ml) (56) |
| *Hygrophoropsis aurantiaca* (Wulfen) Maire | EC_50_ 1.20 (mg/ml) (DPPH) (29) |
| *Hygrophorus eburneus* (Bull.) Fr. | IC_50_ 0.102 mg/ml (DPPH) (57) |
| *Hymenopellis radicata* (Relhan) R.H. Petersen | IC_50_ 1.08 mg/ml (this study) |
| *Hypholoma capnoides* (Fr.) P. Kumm. | EC_50_ 20.85 (mg/ml) (DPPH) (29) |
| *Hypholoma fasciculare* (Huds.) P. Kumm. | EC_50_ 1.13 mg/ml (DPPH) (**Error! Reference source not found.**)  IC_50_ 0.05 mg/ml (this study) |
| *Hypholoma lateritium* (Schaeff.) P. Kumm. | IC_50_ ca. 0.5 – 1.7 mg/ml (DPPH) (58) |
| *Hypsizygus ulmarius* (Bull.) Redhead | IC_50_ 0.120 mg/ml (ABTS) and 0.44 mg/ml (DPPH) (59)  IC_50_ 0.007– 0.008 mg/ml (DPPH) (60) |
| *Infundibulicybe geotropa* (Bull. ex DC.) Harmaja | scavenging activity 5.30 – 12.93 % (DPPH), % 14.77 – 50.39 (ABTS) (6)  scavenging activity 80% (DPPH) (61) |
| *Laccaria amethystina* Cooke | EC_50_ 15.72 (mg/ml) (DPPH) (29) |
| *Laccaria laccata* (Scop.) Cooke | EC_50_ 21.95 (mg/ml) (DPPH) (29) |
| *Lactarius deliciosus* (L.) Gray | IC_50_ 0.494 mg/ml (DPPH) (standard 0.006 mg/ml) (62)  IC_50_ ˃17 mg/ml (DPPH) (63)  EC_50_ 16.31–20.54 mg/ml (DPPH) (24) |
| *Lactifluus piperatus* (L.) Roussel | IC_50_ 0.099 – 0.173 mg/ml (DPPH) (16)  EC_50_ 5.19 – 20.24 mg/ml (DPPH) (64)  IC_50_ 1.40 –1.51 mg/ml (DPPH) 0.52 – 0.60 mg/ml (ABTS) (65)  IC_50_ 0.157 mg/ml (DPPH) (standard Trolox 0.111 mg/ml) (66) |
| *Lactifluus vellereus* (Fr.) Kuntze | IC_50_ 0.68 mg/ml (DPPH) (67)  EC_50_ 7.76 – 17.46 mg/ml (DPPH) (36) |
| *Lactifluus volemus* (Fr.) Kuntze | EC_50_ 21.68 mg/ml (DPPH) (13) |
| *Lentinus lepideus* (Fr.) Fr. | scavenging activity 8.29 – 75.72% (DPPH) (standard BHA 85.25 – 98.74%) (68)  EC_50_ >1 mg/ml (DPPH) (standard Trolox 0.014, ascorbic acid 0.006 mg/ml) (69) |
| *Lentinus sajor-caju* (Fr.) Fr. | scavaging activity 6.40 – 13.65 % (DPPH), 9.73 – 68.97 % (ABTS) (6)  scavaging activity 56.36% (DPPH), 51.41% (ABTS) (70)  12.5 mg/ml (DPPH) (71) |
| *Lentinus tigrinus* (Bull.) Fr. | EC_50_ DPPH >1 mg/ml, (Trolox 0.014, ascorbic acid 0.006 mg/ml) (69)  IC_50_ 0.68 mg/ml (this study) |
| *Lepista nuda* (Bull.) Cooke | EC_50_ 4.41 mg/ml (DPPH) (**Error! Reference source not found.**)  EC_50_ 3.67-8.73 mg/ml (DPPH) (72)  scavaging activity 91.3% (DPPH) (standard BHA 97.4%) (73) |
| *Leucoagaricus leucothites* (Vittad.) Wasser | IC_50_ 8.3 mg/ml (DPPH) (74)  EC_50_ 7.3±0.9 mg/ml (DPPH) (**Error! Reference source not found.**)  IC_50_ 0.35 mg/ml (this study) |
| *Leucopaxillus giganteus* (Sowerby) Singer | IC_50_ 2 mg/ml (DPPH)(polysaccharids) (75)  EC_50_ 3.2 – 3.6 mg/ml (DPPH) (76) |
| *Macrocybe gigantea* (Massee) Pegler & Lodge | EC_50_ 0.096 – 0.108 mg/ml (DPPH) (77)  IC_50_ 0.75 mg/ml (DPPH) (78) |
| *Macrolepiota excoriata* (Schaeff.) Wasser | EC_50_ 1.32 mg/ml (DPPH) (30)  scavaging activity 3.40 % (DPPH), 15.20 % (ABTS) (6) |
| *Macrolepiota mastoidea* (Fr.) Singer | EC_50_ 8.18 – 8.49 mg/ml (DPPH?) (24)  EC_50_ 8.18 – 25.60 mg/ml (DPPH) (79)  EC_50_ 5.4 mg/ml (DPPH) (27) |
| *Macrolepiota procera* (Scop.) Singer | IC_50_ 0.311 mg/ml (DPPH) (standard 0.006 mg/ml) (62)  EC_50_ 5.38 – 6.95 mg/ml (DPPH?) (24)  EC_50_ 4.9 – 7.9 mg/ml (DPPH) (80)  IC_50_ 1.19 – 1.31 mg/ml (DPPH) (19)  EC_50_ 7.22 mg/ml (DPPH) (81)  IC_50_ 0.19 mg/ml (DPPH) (standard BHT 0.09) (82) |
| *Marasmiellus peronatus* (Bolton) J.S. Oliveira | EC_50_ 0.61 mg/ml (83) |
| *Marasmius oreades* (Bolton) Fr. | IC_50_ 0.09 mg/ml (DPPH) (standard GA 0.002 mg/ml) (84)  EC_50_ 3 – 4 mg/ml (DPPH) (85)  IC_50_ 3.54 mg/ml (DPPH) (86)  EC_50_ 2.78 mg/ml (DPPH) (87) |
| *Melanoleuca exscissa* (Fr.) Singer | IC_50_ 0.25 mg/ml (this study) |
| *Mycenastrum corium* (Guers.) Desv. | IC_50_ 0.007 mg/ml (ABTS) (standard Trolox 2.15 mg/ml) (88) |
| *Omphalotus olearius* (DC.) Singer | scavenging activity 22.97 – 60.25 % (DPPH), 48.38 – 88.01 % (ABTS) (6)  EC_50_ 0.9 ± 0.1 mg/ml (DPPH) (**Error! Reference source not found.**) |
| *Panus conchatus* (Bull.) Fr. | EC_50_ DPPH >1 mg/ml (standard Trolox 0.014 mg/ml, ascorbic acid 0.006 mg/ml) (69) |
| *Paralepista flaccida* (Sowerby) Vizzini | EC_50_ >1 mg/ml (89) |
| *Paxillus involutus* (Batsch) Fr. | IC_50_ 0.38 mg/ml (DPPH) and 0.92 mg/ml (ABTS) (a polysaccharide)(90)  IC_50_ 0.016 mg/ml (DPPH) (91)  IC_50_ 0.6 mg/ml (DPPH) (92) |
| *Phaeomarasmius erinaceus* (Fr.) Scherff. ex Romagn. | IC_50_ 2.36 mg/ml (DPPH) (a heterogalactan) (93) |
| *Pholiota adiposa* (Batsch) P. Kumm. | scavenging activity 75.2% (DPPH) (a polysaccharide, standard BHT 65.8%) (94)  IC_50_ 0.25 mg/ml (this study) |
| *Phyllotopsis nidulans* (Pers.) Singer | scavenging activity 49% (DPPH) and 8% (ABTS) (95) |
| *Pleurotus calyptratus* (Lindblad ex Fr.) Sacc. | scavenging activity 12.5% – 23.8% (DPPH) (96) |
| *Pleurotus cornucopiae* (Paulet) Rolland | scavenging activity 12.1% – 29.2% (DPPH) (96)  IC_50_ 0.41 mg/ml (this study) |
| *Pleurotus djamor* (Rumph. ex Fr.) Boedijn | scavenging activity 3.92 – 9.06 % (DPPH), 21.68 – 81.71 % (ABTS) (6)  scavaging activity 8.7% – 22.5 (DPPH) (96)  EC_50_ 0.6 mg/ml (DPPH) (97)  EC_50_ 2.75 mg/ml (DPPH) (98) |
| *Pleurotus dryinus* (Pers.) P. Kumm. | scavaging activity 23.8% – 75.5% (DPPH) (96) |
| *Pleurotus eryngii* (DC.) Quél. | IC_50_ca. 50 mg/ml (DPPH) (48)  EC_50_ 2.67 mg/ml (DPPH) (99)  EC_50_ 1.08 – 1.30 mg/ml (DPPH) (standard cathechin 0.01 mg/ml) (100)  EC_50_ 7.34 mg/ml (DPPH) (101)  scavaging 37.3% – 39.1% (DPPH) (102)  IC_50_0.24 mg/ml (this study) |
| *Pleurotus fossulatus* (Cooke) Sacc. | scavaging 33.6% – 40.6% (DPPH) (103) |
| *Pleurotus nebrodensis* (Inzenga) Quél. | EC_50_ 6.7 ± 0.9 mg/ml (DPPH) (**Error! Reference source not found.**)  scavaging 44.54% – 91.38 (DPPH) (standard BHT 85.25–98.23%) (104) |
| *Pleurotus ostreatus* (Jacq.) P. Kumm. | EC_50_ 13 – 25 mg/ml (DPPH) (45)  IC_50_ 11 mg/ml? (DPPH) (71)  scavaging activity 8.1% – 25.2% (DPPH) (96)  EC_50_ 4.42 mg/ml (DPPH) (101)  IC_50_ 8 mg/ml (hydroxyl and superoxide radicals) (105)  scavaging 87.29% (ABTS) (standard 73.11%) (106)  IC_50_ 0.46 mg/ml (this study) |
| *Pleurotus pulmonarius* (Fr.) Quél. | EC_50_ 0.51 mg/ml (DPPH) and 0.03 mg/ml (ABTS) (107)  EC_50_ 1.21 mg/ml (DPPH) (99)  IC_50_ 8 mg/ml (DPPH) (standard quercetin 0.09 mg/ml) (108)  scavaging activity 7.4% – 27.5% (DPPH) (96) |
| *Pluteus cervinus* (Schaeff.) P. Kumm. | 25.6% – 92.7% (DPPH) (standard BHT 96.19% – 96.97% and 75.6 – 95.1% for OH scavaging) (109)  IC_50_ 0.11 mg/ml (this study) |
| *Psathyrella bivelata* Contu | 0.27 mg/ml (this study) |
| *Psathyrella candolleana* (Fr.) Maire | IC_50_ 0.0015 mg/ml (DPPH) (a flavonoid) (110)  scavaging 89.76% (DPPH) (111) |
| *Rhodotus palmatus* (Bull.) Maire | EC_50_ 7.58 – 15.48 mg/ml (DPPH) (36) |
| *Russula alutacea* (Pers.) Fr. | EC_50_ 0.85 – 1.25 mg/ml (DPPH) (polysaccharides, standard vit. C 0.55 mg/ml) (112)  EC_50_ 0.38 mg/ml (polysaccharides, standard vit. C 0.30 mg/ml) (DPPH) (113) |
| *Russula anthracina* Romagn. | 90.62% (DPPH) (standard BHT 98.24%) (114) |
| *Russula cyanoxantha* (Schaeff.) Fr. | IC_50_ 0.086 – 0.262 mg/ml (DPPH) (16)  IC_50_ 0.835 mg/ml (DPPH) (115)  IC_50_ 0.69 mg/ml (DPPH) (30)  IC_50_ 0.073 mg/ml (ABTS) 0.866 (DPPH) (standard BHT 0.051 mg/ml) (116) |
| *Russula delica* Fr. | EC_50_ 20.53 (mg/ml) (DPPH) (29)  IC_50_ 44.0 mg/ml (standards BHA, BHT, α -Tocopherol < 0.1 mg/ml) (DPPH) (117) |
| *Russula emetica* (Schaeff.) Pers. | EC_50_ 1.13 mg/ml (DPPH) (standard Trolox 0.04 mg/ml) (56) |
| *Russula emeticolor* J. Schaeffer | IC_50_ 0.12 mg/ml (this study) |
| *Russula integra* (L.) Fr. | IC_50_ 1.720 mg/ml (TBARS assay) (standard Trolox 19.6 mg/ml) (118) |
| *Russula nigricans* Fr. | IC_50_ 0.12 mg/ml (TBARS assay) (standard Trolox 0.019 mg/ml) (118) |
| *Russula olivacea* Pers. | IC_50_ 1.47 mg/ml (DPPH) (30) |
| *Russula rosea* Pers. | IC_50_ 0.092 mg/ml (TBARS assay) (standard Trolox 0.019 mg/ml) (118)  scavenging activity 87.85% at 2.0 mg/ml (DPPH) (standard BHT 96.97%) (119) |
| *Russula virescens* (Schaeff.) Fr. | EC_50_ 30.21 mg/ml (DPPH) (14)  IC_50_ 1.48 mg/ml (polysaccharides) (ABTS) (120)  IC_50_ 0.44 mg/ml (ABTS) 0.22 mg/ml (DPPH) (121)  IC_50_ 15 mg/ml (DPPH) (122) |
| *Russula xerampelina* (Schaeff.) Fr. | scavenging activity 31.2% (DPPH) (123) |
| *Strobilurus esculentus* (Wulfen) Singer | EC_50_ 29.6 mg/ml (DPPH) (standard vit. C 21.8 mg/ml) (124) |
| *Tricholoma acerbum* (Bull.) Quél. | EC_50_ 3.60 mg/ml (DPPH) (**Error! Reference source not found.**) |
| *Tricholoma equestre* (L.) P. Kumm. | IC_50_ 1.5 mg/ml (DPPH) (standard vit. C 0.003) (125)  EC_50_ 0.035 mg/ml (DPPH) (standard BHA 0.025) (126)  EC_50_ 3.60 mg/ml (DPPH) (127) |
| *Tricholoma sulphureum* (Bull.) P. Kumm. | EC_50_ 4.69 (mg/ml) (DPPH) (29) |
| *Tricholoma terreum* (Schaeff.) P. Kumm. | IC_50_ 12.17 mg/ml (DPPH) (standard BHA 0.32 mg/ml) (128)  24.6% (DPPH) and 88.4% (ABTS) (standard BHA 95.9%, 96%) (129)  IC_50_ 31.42 mg/ml (DPPH) (standard GA 0.037 mg/ml) (130) |
| *Tricholoma ustale* (Fr.) P. Kumm. | EC_50_ 4.89 mg/ml (DPPH) (13) |
| *Tubaria furfuracea* (Pers.) Gillet | IC_50_ 74.15 mg of dry extract per mg DPPH (DPPH) (standard Trolox 0.134 mg/mg) (54) |
| *Volvariella bombycina* (Schaeff.) Singer | scavenging activity 18% (DPPH) (standard vit. C 95%) (131) |
| *Volvariella volvacea* (Bull.) Singer | IC_50_ 2.05 mg/ml (DPPH) (standard vit. C 0.09 mg/ml) (132)  scavenging activity 82.9% (DPPH) (free phenolic extracts) (133) |
| *Volvopluteus gloiocephalus* (DC.) Vizzini, Contu & Justo | IC_50_ 3.60 mg/ml (DPPH) (standard GA 0.037) (130) |
| *Xerula pudens* (Pers.) Singer | IC_50_ 0.23 mg/ml (this study) |

1. Gąsecka, M.; Magdziak, Z.; Siwulski, M.; Mleczek, M. Profile of phenolic and organic acids, antioxidant properties and ergosterol content in cultivated and wild growing species of *Agaricus*. *European Food Research and Technology* 2018, *244*, 259-268.

2. Barros, L.; Venturini, B.A.; Baptista, P.; Estevinho, L.M.; Ferreira, I.C. Chemical composition and biological properties of Portuguese wild mushrooms: a comprehensive study. *Journal of agricultural and food chemistry* 2008, *56*, 3856-3862.

3. Öztürk, M.; Duru, M.E.; Kivrak, Ş.; Mercan-Doğan, N.; Türkoglu, A.; Özler, M.A. In vitro antioxidant, anticholinesterase and antimicrobial activity studies on three *Agaricus* species with fatty acid compositions and iron contents: A comparative study on the three most edible mushrooms. *Food and Chemical Toxicology* 2011, *49*, 1353-1360.

4. Jagadish, L.K.; Krishnan, V.V.; Shenbhagaraman, R.; Kaviyarasan, V. Comparitive study on the antioxidant, anticancer and antimicrobial property of *Agaricus bisporus* (JE Lange) Imbach before and after boiling. *African Journal of Biotechnology* 2009, *8*.

5. Glamočlija, J.; Stojković, D.; Nikolić, M.; Ćirić, A.; Reis, F.S.; Barros, L.; Ferreira, I.C.; Soković, M. A comparative study on edible *Agaricus* mushrooms as functional foods. *Food & function* 2015, *6*, 1900-1910.

6. Kalyoncu, F.; Oskay, M.; Kayalar, H. Antioxidant activity of the mycelium of 21 wild mushroom species. *Mycology* 2010, *1*, 195-199.

7. Kosanic, M.; Ranković, B.; Rančič, A.; Stanojkovic, T. Evaluation of metal contents and bioactivity of two edible mushrooms *Agaricus campestris* and *Boletus edulis*. *Emirates Journal of Food and Agriculture* 2017.

8. Soares, A.A.; de Souza, C.G.M.; Daniel, F.M.; Ferrari, G.P.; da Costa, S.M.G.; Peralta, R.M. Antioxidant activity and total phenolic content of *Agaricus brasiliensis* (*Agaricus blazei* Murril) in two stages of maturity. *Food chemistry* 2009, *112*, 775-781.

9. Tsai, S.-Y.; Tsai, H.-L.; Mau, J.-L. Antioxidant properties of *Agaricus blazei, Agrocybe cylindracea*, and *Boletus edulis*. *LWT-Food Science and Technology* 2007, *40*, 1392-1402.

10. Carneiro, A.A.; Ferreira, I.C.; Dueñas, M.; Barros, L.; Da Silva, R.; Gomes, E.; Santos-Buelga, C. Chemical composition and antioxidant activity of dried powder formulations of *Agaricus blazei* and *Lentinus edodes*. *Food chemistry* 2013, *138*, 2168-2173.

11. Al-Fatimi, M.; Schröder, G.; Kreisel, H.; Lindequist, U. Biological activities of selected basidiomycetes from Yemen. *Die Pharmazie-An International Journal of Pharmaceutical Sciences* 2013, *68*, 221-226.

12. KARTAL, D.İ.; ŞAYAK, O.; İsmail, A. Investigation of antioxidant activities of *Agrocybe praecox* fungus. *Mantar Dergisi 13*, 37-43.

13. Reis, F.S.; Heleno, S.A.; Barros, L.; Sousa, M.J.; Martins, A.; Santos‐Buelga, C.; Ferreira, I.C. Toward the antioxidant and chemical characterization of mycorrhizal mushrooms from Northeast Portugal. *Journal of Food Science* 2011, *76*, C824-C830.

14. Leal, A.R.; Barros, L.; Barreira, J.C.; Sousa, M.J.; Martins, A.; Santos-Buelga, C.; Ferreira, I.C. Portuguese wild mushrooms at the “pharma–nutrition” interface: Nutritional characterization and antioxidant properties. *Food Research International* 2013, *50*, 1-9.

15. Zhu, Y.; Ding, X.; Wang, M.; Hou, Y.; Hou, W.; Yue, C. Structure and antioxidant activity of a novel polysaccharide derived from *Amanita caesarea*. *Molecular Medicine Reports* 2016, *14*, 3947-3954.

16. Kosanic, M.; Rankovic, B.; Dasic, M. Antioxidant and antimicrobial properties of mushrooms. *Bulgarian Journal of Agricultural Science* 2013, *19*, 1040-1046.

17. Paloi, S.; Acharya, K. Evaluation of antioxidative activity and chemical composition of ethanolic extract from *Amanita vaginata* (Bull.) Lam.: an in vitro study. *Asian J Pharm Clin Res* 2014, *7*, 88-92.

18. Lung, M.-Y.; Chang, Y.-C. Antioxidant properties of the edible basidiomycete *Armillaria mellea* in submerged cultures. *International Journal of Molecular Sciences* 2011, *12*, 6367-6384.

19. Erbiai, E.H.; da Silva, L.P.; Saidi, R.; Lamrani, Z.; Esteves da Silva, J.C.; Maouni, A. Chemical composition, bioactive compounds, and antioxidant activity of two wild edible mushrooms *Armillaria mellea* and *Macrolepiota procera* from two countries (Morocco and Portugal). *Biomolecules* 2021, *11*, 575.

20. Strapáč, I.; Baranová, M.; Smrčová, M.; Bedlovičová, Z. Antioxidant activity of honey mushrooms (*Armillaria mellea*). *Folia Veterinaria* 2016, *60*, 37-41.

21. Gao, L.W.; Wang, J.W. Antioxidant potential and DNA damage protecting activity of aqueous extract from *Armillaria mellea*. *Journal of Food Biochemistry* 2012, *36*, 139-148.

22. Vaz, J.A.; Barros, L.; Martins, A.; Santos-Buelga, C.; Vasconcelos, M.H.; Ferreira, I.C. Chemical composition of wild edible mushrooms and antioxidant properties of their water soluble polysaccharidic and ethanolic fractions. *Food Chemistry* 2011, *126*, 610-616.

23. Ebrahimzadeh, M.A.; Nabavi, S.M.; Nabavi, S.F.; Eslami, S. Antioxidant and free radical scavenging activities of culinary-medicinal mushrooms, golden chanterelle *Cantharellus cibarius* and Angel's wings *Pleurotus porrigens*. *International Journal of Medicinal Mushrooms* 2010, *12*.

24. Froufe, H.J.; Abreu, R.; Ferreira, I.C. A QCAR model for predicting antioxidant activity of wild mushrooms. *SAR and QSAR in Environmental Research* 2009, *20*, 579-590.

25. Sevindik, M. Wild edible mushroom *Cantharellus cibarius* as a natural antioxidant food. *Turkish Journal of Agriculture-Food Science and Technology* 2019, *7*, 1377-1381.

26. Pereira, E.; Barros, L.; Martins, A.; Ferreira, I.C. Towards chemical and nutritional inventory of Portuguese wild edible mushrooms in different habitats. *Food Chemistry* 2012, *130*, 394-403.

27. Ćirić, A.; Kruljević, I.; Stojković, D.; Fernandes, Â.; Barros, L.; Calhelha, R.C.; Ferreira, I.C.; Soković, M.; Glamočlija, J. Comparative investigation on edible mushrooms *Macrolepiota mastoidea, M. rhacodes* and *M. procera*: Functional foods with diverse biological activities. *Food & Function* 2019, *10*, 7678-7686.

28. Kosanić, M.; Petrović, N.; Stanojković, T. Bioactive properties of *Clitocybe geotropa* and *Clitocybe nebularis*. *Journal of Food Measurement and Characterization* 2020, *14*, 1046-1053.

29. Heleno, S.A.; Barros, L.; Sousa, M.J.; Martins, A.; Ferreira, I.C. Tocopherols composition of Portuguese wild mushrooms with antioxidant capacity. *Food Chemistry* 2010, *119*, 1443-1450.

30. Grangeia, C.; Heleno, S.A.; Barros, L.; Martins, A.; Ferreira, I.C. Effects of trophism on nutritional and nutraceutical potential of wild edible mushrooms. *Food Research International* 2011, *44*, 1029-1035.

31. Sharma, S.K.; Gautam, N. Chemical, bioactive, and antioxidant potential of twenty wild culinary mushroom species. *BioMed Research International* 2015, *2015*.

32. Novaković, A.R.; Karaman, M.A.; Kaišarević, S.N.; Belović, M.M.; Radusin, T.I.; Beribaka, M.B.; Ilić, N.M. *Coprinellus disseminatus* (Pers.) JE Lange 1938: In vitro antioxidant and antiproliferative effects. *Food and Feed Research* 2016, *43*, 93-101.

33. Nguyen, T.K.; Lee, M.W.; Yoon, K.N.; Kim, H.Y.; Jin, G.-H.; Choi, J.-H.; Im, K.H.; Lee, T.S. In vitro antioxidant, anti-diabetic, anti-cholinesterase, tyrosinase and nitric oxide inhibitory potential of fruiting bodies of *Coprinellus micaceus*. *Journal of Mushroom* 2014, *12*, 330-340.

34. Dundar, A.; Okumus, V.; Ozdemir, S.; Celik, K.S.; Boğa, M.; Ozcagli, E. Determination of cytotoxic, anticholinesterase, antioxidant and antimicrobial activities of some wild mushroom species. *Cogent Food & Agriculture* 2016, *2*, 1178060.

35. Tešanović, K.; Pejin, B.; Šibul, F.; Matavulj, M.; Rašeta, M.; Janjušević, L.; Karaman, M. A comparative overview of antioxidative properties and phenolic profiles of different fungal origins: fruiting bodies and submerged cultures of *Coprinus comatus* and *Coprinellus truncorum*. *Journal of food science and technology* 2017, *54*, 430-438.

36. Heleno, S.A.; Barros, L.; Martins, A.; Queiroz, M.J.R.; Santos-Buelga, C.; Ferreira, I.C. Phenolic, polysaccharidic, and lipidic fractions of mushrooms from Northeastern Portugal: chemical compounds with antioxidant properties. *Journal of Agricultural and Food Chemistry* 2012, *60*, 4634-4640.

37. Yadav, M.; Yadav, A.; Yadav, J.P. In vitro antioxidant activity and total phenolic content of endophytic fungi isolated from *Eugenia jambolana* Lam. *Asian Pacific journal of tropical medicine* 2014, *7*, S256-S261.

38. Tibuhwa, D.D.; Lyantagaye, S.L.; Mshandete, A.M. Effect of different post-harvest treatments on nutritive and antioxidant activities of wild edible *Coprinus cinereus* (Schaeff.) S. Gray from Tanzania. *Int J Res Biol Sci* 2012, *2*, 150-156.

39. Stanojković, T.; Kosanić, M. Biomedica potential of selected mushroom species. *CONTEMPORARY MATERIALS 11*.

40. Li, B.; Lu, F.; Suo, X.; Nan, H.; Li, B. Antioxidant properties of cap and stipe from *Coprinus comatus*. *Molecules* 2010, *15*, 1473-1486.

41. TSAI, S.Y.; TSAI, H.L.; MAU, J.L. Antioxidant properties of *Coprinus comatus*. *Journal of food biochemistry* 2009, *33*, 368-389.

42. Kosanić, M.; Ranković, B.; Stanojković, T.; Radović-Jakovljević, M.; Ćirić, A.; Grujičić, D.; Milošević-Djordjević, O. *Craterellus cornucopioides* edible mushroom as source of biologically active compounds. *Natural Product Communications* 2019, *14*, 1934578X19843610.

43. Sevim, K.; BOSTANCI, A.; KOCABAŞ, A.; Yasin, U.; Gökhan, S. Cell growth inhibitory potential of *Craterellus cornucopioides* (L.) Pers. together with antioxidant and antimicrobial properties. *Anatolian Journal of Botany* 2018, *2*, 60-64.

44. Vamanu, E.; Voica, A. Total phenolic analysis, antimicrobial and antioxidant activity of some mushroom tinctures from medicinal and edible species, by in vitro and in vivo tests. *Scientific Bulletin. Series F. Biotechnologies* 2017, *21*, 318-324.

45. Cilerdzic, J.; Stajic, M.; Vukojevic, J.; Milovanovic, I.; Muzgonja, N. Antioxidant and antifungal potential of *Pleurotus ostreatus* and *Agrocybe cylindracea* basidiocarps and mycelia. *Current Pharmaceutical Biotechnology* 2015, *16*, 179-186.

46. Landingin, H.; Francisco, B.; Dulay, R.; Kalaw, S.; Reyes, R. Mycochemical screening, proximate nutritive composition and radical scavenging activity of *Cyclocybe cylindracea* and *Pleurotus cornucopiae*. *Journal of Fungal Biology* 2021, *11*, 37-50.

47. Keller, C.; Maillard, M.; Keller, J.; Hostettmann, K. Screening of European fungi for antibacterial, antifungal, larvicidal, molluscicidal, antioxidant and free-radical scavenging activities and subsequent isolation of bioactive compounds. *Pharmaceutical biology* 2002, *40*, 518-525.

48. Butkhup, L.; Samappito, W.; Jorjong, S. Evaluation of bioactivities and phenolic contents of wild edible mushrooms from northeastern Thailand. *Food science and biotechnology* 2018, *27*, 193-202.

49. Zhang, Z.; Lv, G.; He, W.; Shi, L.; Pan, H.; Fan, L. Effects of extraction methods on the antioxidant activities of polysaccharides obtained from *Flammulina velutipes*. *Carbohydrate polymers* 2013, *98*, 1524-1531.

50. Shah, S.; Ukaegbu, C.; Hamid, H.; Alara, O. Evaluation of antioxidant and antibacterial activities of the stems of *Flammulina velutipes* and *Hypsizygus tessellatus* (white and brown var.) extracted with different solvents. *Journal of Food Measurement and Characterization* 2018, *12*, 1947-1961.

51. Ukaegbu, C.; Shah, S.; Hazrulrizawati, A.; Alara, O. Acetone extract of *Flammulina velutipes* caps: A promising source of antioxidant and anticancer agents. *Beni-Suef University journal of basic and applied sciences* 2018, *7*, 675-682.

52. Ukaegbu, C.I.; Shah, S.R.; Hamid, H.A.; Alara, O.R.; Sarker, M.; Islam, Z. Phenolic compounds of aqueous and methanol extracts of *Hypsizygus tessellatus* (brown and white var.) and *Flammulina velutipes* caps: Antioxidant and antiproliferative activities. *Pharmaceutical Chemistry Journal* 2020, *54*, 170-183.

53. Zhang, Z.; Jin, Q.; Lv, G.; Fan, L.; Pan, H.; Fan, L. Comparative study on antioxidant activity of four varieties of *Flammulina velutipes* with different colour. *International journal of food science & technology* 2013, *48*, 1057-1064.

54. Nowacka, N.; Nowak, R.; Drozd, M.; Olech, M.; Los, R.; Malm, A. Antibacterial, antiradical potential and phenolic compounds of thirty-one polish mushrooms. *PloS one* 2015, *10*, e0140355.

55. Ragupathi, V.; Stephen, A.; Arivoli, D.; Kumaresan, S. Antibacterial activity, in vitro antioxidant potential and gc-MS characterization of methanolic extract of *Gymnopilus junonius*, a wild mushroom from Southern Western Ghats, India. *Eur. J. Biomed* 2018, *5*, 650-657.

56. Reis, F.S.; Pereira, E.; Barros, L.; Sousa, M.J.; Martins, A.; Ferreira, I.C. Biomolecule profiles in inedible wild mushrooms with antioxidant value. *Molecules* 2011, *16*, 4328-4338.

57. Kosanic, M.M.; Šeklic, D.S.; Jovanovic, M.M.; Petrovic, N.N.; Markovic, S.D. *Hygrophorus eburneus*, edible mushroom, a promising natural bioactive agent. *EXCLI journal* 2020, *19*, 442.

58. Li, H.; Nam, W.-S.; Moon, B.; Lee, C. Antioxidant activity and phenolic content of brick caps mycelium (*Naematoloma sublateritium*) extracts. *Food Science and Biotechnology* 2014, *23*, 1425-1431.

59. Greeshma, P.; Ravikumar, K.S.; Neethu, M.N.; Pandey, M.; Zuhara, K.F.; Janardhanan, K.K. Antioxidant, anti-inflammatory, and antitumor activities of cultured mycelia and fruiting bodies of the elm oyster mushroom, *Hypsizygus ulmarius* (Agaricomycetes). *International journal of medicinal mushrooms* 2016, *18*.

60. Al-Faqeeh, L.A.S.; Naser, R.; Kagne, S. Phytochemical screening and antioxidant activity of *Hypsizygus ulmarius* (Bull.). *Research J. Pharm. and Tech* 2020, *13*, 4297-4302.

61. Dizeci, N.; Onar, O.; Karaca, B.; Demirtas, N.; Coleri Cihan, A.; Yildirim, O. Comparison of the chemical composition and biological effects of *Clitocybe nebularis* and *Infundibulicybe geotropa*. *Mycologia* 2021, *113*, 1156-1168.

62. Kosanić, M.; Ranković, B.; Rančić, A.; Stanojković, T. Evaluation of metal concentration and antioxidant, antimicrobial, and anticancer potentials of two edible mushrooms *Lactarius deliciosus* and *Macrolepiota procera*. *Journal of food and drug analysis* 2016, *24*, 477-484.

63. Onbaşili, D.; Çelik, G.; Katırcıoğlu, H.; Narin, I. Antimicrobial, antioxidant activities and chemical composition of *Lactarius deliciosus* (L.) collected from Kastamonu province of Turkey. *Kastamonu University Journal of Forestry Faculty* 2015, *15*, 98-103.

64. Barros, L.; Baptista, P.; Ferreira, I.C. Effect of *Lactarius piperatus* fruiting body maturity stage on antioxidant activity measured by several biochemical assays. *Food and chemical Toxicology* 2007, *45*, 1731-1737.

65. Bera, I.; Datta, B.; Das, K.; Seal, T. Effect of solvent extraction system on the antioxidant properties of seven wild edible mushrooms and identification of phenolic compounds by high-performance liquid chromatography. *Pharmacognosy Magazine* 2021, *17*, 180.

66. Ao, T.; Deb, C.R. Nutritional and antioxidant potential of some wild edible mushrooms of Nagaland, India. *Journal of Food Science and Technology* 2019, *56*, 1084-1089.

67. Dogan, H.H.; Aydin, S. Some biological activities of Lactarius vellereus (Fr.) Fr. in Turkey. *Pakistan Journal of Biological Sciences* 2013, *16*, 1279-1286.

68. Yoon, K.N.; Alam, N.; Lee, K.R.; Shin, P.G.; Cheong, J.C.; Yoo, Y.B.; Lee, T.S. Antioxidant and antityrosinase activities of various extracts from the fruiting bodies of *Lentinus lepideus*. *Molecules* 2011, *16*, 2334-2347.

69. Macáková, K.; Opletal, L.; Polášek, M.; Samková, V. Free-radical scavenging activity of some European Polyporales. *Natural Product Communications* 2010, *5*, 1934578X1000500623.

70. Singdevsachan, S.K.; Patra, J.K.; Thatoi, H. Nutritional and bioactive potential of two wild edible mushrooms (*Lentinus sajor-caju* and *Lentinus torulosus*) from Similipal Biosphere Reserve, India. *Food Science and Biotechnology* 2013, *22*, 137-145.

71. Chirinang, P.; Intarapichet, K.-O. Amino acids and antioxidant properties of the oyster mushrooms, *Pleurotus ostreatus* and *Pleurotus sajor-caju*. *Science Asia* 2009, *35*, 326-331.

72. Pinto, S.; Barros, L.; Sousa, M.J.; Ferreira, I.C. Chemical characterization and antioxidant properties of *Lepista nuda* fruiting bodies and mycelia obtained by in vitro culture: Effects of collection habitat and culture media. *Food research international* 2013, *51*, 496-502.

73. Elmastas, M.; Isildak, O.; Turkekul, I.; Temur, N. Determination of antioxidant activity and antioxidant compounds in wild edible mushrooms. *Journal of Food Composition and Analysis* 2007, *20*, 337-345.

74. Aslim, B.; Ozturk, S. Phenolic composition and antimicrobial and antioxidant activities of *Leucoagaricus leucothites* (Vittad.) Wasser. *Journal of Medicinal Food* 2011, *14*, 1419-1424.

75. Bao, Y.; Ma, Y.; Li, Y.; Zhang, Y.; Wu, Y.; Liu, C. Structure identification and free radical scavenging activity of polysaccharide from fruit bodies of *Leucopaxillus giganteus*. *Food Sci* 2016, *37*, 71-76.

76. Barros, L.; Ferreira, I.C.; Baptista, P. Phenolics and antioxidant activity of mushroom *Leucopaxillus giganteus* mycelium at different carbon sources. *Food Science and Technology International* 2008, *14*, 47-55.

77. Gaur, T.; Rao, P. Antioxidant potential of the giant mushroom, *Macrocybe gigantea* (Agaricomycetes), from India in different drying methods. *International Journal of Medicinal Mushrooms* 2016, *18*.

78. Pushpa, H.; Anand, M.; Kasimaiah, P.; Pradeep, P.; Purushothama, K. Antioxidant and anticancer activity of *Tricholoma giganteum* Massee an edible wild mushroom. *Acad J Can Res* 2014, *7*, 146-151.

79. Barros, L.; Baptista, P.; Correia, D.M.; Sá Morais, J.; Ferreira, I.C. Effects of conservation treatment and cooking on the chemical composition and antioxidant activity of Portuguese wild edible mushrooms. *Journal of Agricultural and Food Chemistry* 2007, *55*, 4781-4788.

80. Fernandes, Â.; Barros, L.; Barreira, J.C.; Antonio, A.L.; Oliveira, M.B.P.; Martins, A.; Ferreira, I.C. Effects of different processing technologies on chemical and antioxidant parameters of *Macrolepiota procera* wild mushroom. *LWT-Food Science and Technology* 2013, *54*, 493-499.

81. Witkowska, A.M.; Zujko, M.E.; Mironczuk-Chodakowska, I. Comparative study of wild edible mushrooms as sources of antioxidants. *International Journal of Medicinal Mushrooms* 2011, *13*.

82. AYTAR, E.C.; AKATA, İ.; Leyla, A. Antioxidant and antimicrobial activities of *Armillaria Mellea* and *Macrolepiota Procera* Extracts. *Mantar Dergisi* 2020, *11*, 121-128.

83. Nikolovska Nedelkoska, D.; Tusevski, O.; Rusevska, K.; Gadzovska Simic, S.; Karadelev, M. Phenolic content and antioxidant activity of selected wild mushrooms from Tricholomataceae family, collected in Macedonia. In Proceedings of the Proceedings of the 5th Congress of Ecologists of Macedonia with International Participation, Ohrid, 2016; pp. 19-22.

84. Shomali, N.; Onar, O.; Karaca, B.; Demirtas, N.; Cihan, A.C.; Akata, I.; Yildirim, O. Antioxidant, anticancer, antimicrobial, and antibiofilm properties of the culinary-medicinal fairy ring mushroom, *Marasmius oreades* (Agaricomycetes). *International Journal of Medicinal Mushrooms* 2019, *21*.

85. Vieira, V.; Marques, A.; Barros, L.; Barreira, J.; Ferreira, I.C. Insights in the antioxidant synergistic effects of combined edible mushrooms: phenolic and polysaccharidic extracts of *Boletus edulis* and *Marasmius oreades*. *Journal of Food and Nutrition Research* 2012, *51*, 109-112.

86. Ramesh, C.; Pattar, M.G. Antimicrobial properties, antioxidant activity and bioactive compounds from six wild edible mushrooms of western ghats of Karnataka, India. *Pharmacognosy research* 2010, *2*, 107.

87. Queirós, B.; Barreira, J.C.; Sarmento, A.C.; Ferreira, I.C. In search of synergistic effects in antioxidant capacity of combined edible mushrooms. *International journal of food sciences and nutrition* 2009, *60*, 160-172.

88. Kumlay, A.M.; Kocak, M.Z.; Koyuncu, M.; GÜLLER, U. Bioanalysis of total phenolic contents, volatile compounds, and radical scavenging activities of three wild edible mushrooms. *Studia Universitatis Babes-Bolyai, Chemia* 2021, *66*.

89. Macáková, K. Biological activity of selected taxons of mushrooms from divisions ascomycota and basidiomycota. 2011.

90. Liu, Y.; Zhou, Y.; Liu, M.; Wang, Q.; Li, Y. Extraction optimization, characterization, antioxidant and immunomodulatory activities of a novel polysaccharide from the wild mushroom *Paxillus involutus*. *International Journal of Biological Macromolecules* 2018, *112*, 326-332.

91. Zhang, J.-X.; Lv, J.-H.; Zhao, L.-Q.; Shui, X.-X.; Zhang, J.; Wang, L.-A. Coumarin-pi, a new antioxidant coumarin derivative from *Paxillus involutus*. *Natural Product Research* 2020, *34*, 1246-1249.

92. Reis, F.S.; Ferreira, I.C.; Barros, L.; Martins, A. A comparative study of tocopherols composition and antioxidant properties of in vivo and in vitro ectomycorrhizal fungi. *LWT-Food Science and Technology* 2011, *44*, 820-824.

93. Qu, Y.; Yan, J.; Zhang, X.; Song, C.; Zhang, M.; Mayo, K.H.; Sun, L.; Cheng, H.; Zhou, Y. Structure and antioxidant activity of six mushroom-derived heterogalactans. *International Journal of Biological Macromolecules* 2022, *209*, 1439-1449.

94. Deng, P.; Zhang, G.; Zhou, B.; Lin, R.; Jia, L.; Fan, K.; Liu, X.; Wang, G.; Wang, L.; Zhang, J. Extraction and in vitro antioxidant activity of intracellular polysaccharide by *Pholiota adiposa* SX-02. *Journal of bioscience and bioengineering* 2011, *111*, 50-54.

95. Lee, J.; Hong, J.-H.; Kim, J.-D.; Ahn, B.J.; Kim, B.S.; Kim, G.-H.; Kim, J.-J. The antioxidant properties of solid-culture extracts of basidiomycetous fungi. *The Journal of General and Applied Microbiology* 2013, *59*, 279-285.

96. Jean-Philippe, S.R. Antioxidant properties of some edible fungi in the genus *Pleurotus*. 2005. Thesis, University of Tennessee, Knoxville.

97. Acharya, K.; Khatua, S.; Ray, S. Quality assessment and antioxidant study of *Pleurotus djamor* (Rumph. ex Fr.) Boedijn. *Journal of Applied Pharmaceutical Science* 2017, *7*, 105-110.

98. Sudha, G.; Janardhanan, A.; Moorthy, A.; Chinnasamy, M.; Gunasekaran, S.; Thimmaraju, A.; Gopalan, J. Comparative study on the antioxidant activity of methanolic and aqueous extracts from the fruiting bodies of an edible mushroom *Pleurotus djamor*. *Food science and biotechnology* 2016, *25*, 371-377.

99. Xu, W.W.; Li, B.; Lai, E.T.C.; Chen, L.; Huang, J.J.H.; Cheung, A.L.M.; Cheung, P.C.K. Water extract from *Pleurotus pulmonarius* with antioxidant activity exerts in vivo chemoprophylaxis and chemosensitization for liver cancer. *Nutrition and cancer* 2014, *66*, 989-998.

100. Lin, J.-T.; Liu, C.-W.; Chen, Y.-C.; Hu, C.-C.; Juang, L.-D.; Shiesh, C.-C.; Yang, D.-J. Chemical composition, antioxidant and anti-inflammatory properties for ethanolic extracts from *Pleurotus eryngii* fruiting bodies harvested at different time. *LWT-Food Science and Technology* 2014, *55*, 374-382.

101. Gąsecka, M.; Mleczek, M.; Siwulski, M.; Niedzielski, P. Phenolic composition and antioxidant properties of *Pleurotus ostreatus* and *Pleurotus eryngii* enriched with selenium and zinc. *European Food Research and Technology* 2016, *242*, 723-732.

102. Yildirim, N.C.; Turkoglu, S.; Yildirim, N.; KAPLAN INCE, O. Antioxidant properties of wild edible mushroom *Pleurotus eryngii* collected from Tunceli province of Turkey. *Digest Journal of Nanomaterials & Biostructures (DJNB)* 2012, *7*.

103. Bhatia, P.; Bansal, C.; Prakash, R.; Nagaraja, T. Selenium uptake and associated anti-oxidant properties in *Pleurotus fossulatus* cultivated on wheat straw from seleniferous fields. *Acta Alimentaria* 2014, *43*, 280-287.

104. Alam, N.; Yoon, K.N.; Lee, T.S. Evaluation of the antioxidant and antityrosinase activities of three extracts from *Pleurotus nebrodensis* fruiting bodies. *African Journal of Biotechnology* 2011, *10*, 2978-2986.

105. Jayakumar, T.; Thomas, P.; Geraldine, P. In-vitro antioxidant activities of an ethanolic extract of the oyster mushroom, *Pleurotus ostreatus*. *Innovative Food Science & Emerging Technologies* 2009, *10*, 228-234.

106. Yim, H.; Chye, F.; Tan, C.; Ng, Y.; Ho, C. Antioxidant activities and total phenolic content of aqueous extract of *Pleurotus ostreatus* (cultivated oyster mushroom). *Malaysian Journal of Nutrition* 2010, *16*, 281-291.

107. Contato, A.G.; Inácio, F.D.; de Araújo, C.A.V.; Brugnari, T.; Maciel, G.M.; Haminiuk, C.W.I.; Bracht, A.; Peralta, R.M.; de Souza, C.G.M. Comparison between the aqueous extracts of mycelium and basidioma of the edible mushroom *Pleurotus pulmonarius*: Chemical composition and antioxidant analysis. *Journal of Food Measurement and Characterization* 2020, *14*, 830-837.

108. Abidin, M.H.Z.; Abdullah, N.; Abidin, N.Z. Protective effect of antioxidant extracts from grey oyster mushroom, *Pleurotus pulmonarius* (Agaricomycetes), against human low-density lipoprotein oxidation and aortic endothelial cell damage. *International journal of medicinal mushrooms* 2016, *18*.

109. Nguyen, T.K.; Im, K.H.; Choi, J.; Shin, P.G.; Lee, T.S. Evaluation of antioxidant, anti-cholinesterase, and anti-inflammatory effects of culinary mushroom Pleurotus pulmonarius. *Mycobiology* 2016, *44*, 291-301.

110. Pan, Y.; Zheng, W.; Yang, S. Chemical and activity investigation on metabolites produced by an endophytic fungi *Psathyrella candolleana* from the seed of Ginkgo biloba. *Natural Product Research* 2020, *34*, 3130-3133.

111. Dundar, A.; Okumus, V.; Ozdemir, S.; Celik, K.S.; Boga, M.; Ozcagli, E.; Ozhan, G.; Yildiz, A. Antioxidant, antimicrobial, cytotoxic and anticholinesterase activities of seven mushroom species with their phenolic acid composition. *Journal of Horticulture* 2015, 1-6.

112. Wang, B.; Yang, Q.; Chen, T.; Qin, X.; Tang, Q.; Xiao, Y.; Yang, Y.; Zhao, Y. Preparation and in vitro antioxidant activity of sulfated water-insoluble polysaccharides from *Russula alutacea* Fr. *Southwest China Journal of Agricultural Sciences* 2017, *30*, 2673-2679.

113. Zhao, Y.; Li, X.; Chen, T.; Tang, Q.; Qiu, L.; Wang, B.; Yang, Q. Preparation and antioxidant activity of phosphorylated polysaccharides from *Russula alutacea* Fr. *Ekoloji* 2018, *27*, 17-22.

114. Akata, I.; Ergönül, B.; Kalyoncu, F. Chemical compositions and antioxidant activities of 16 wild edible mushroom species grown in Anatolia. *International Journal of Pharmacology* 2012, *8*, 134-138.

115. Ribeiro, B.; Lopes, R.; Andrade, P.B.; Seabra, R.M.; Gonçalves, R.F.; Baptista, P.; Quelhas, I. Comparative study of phytochemicals and antioxidant potential of wild edible mushroom caps and stipes. *Food chemistry* 2008, *110*, 47-56.

116. Panda, M.K.; Das, S.K.; Mohapatra, S.; Debata, P.R.; Tayung, K.; Thatoi, H. Mycochemical composition, bioactivities, and phylogenetic placement of three wild edible *Russula* species from Northern Odisha, India. *Plant Biosystems-An International Journal Dealing with all Aspects of Plant Biology* 2021, *155*, 1041-1055.

117. Yaltirak, T.; Aslim, B.; Ozturk, S.; Alli, H. Antimicrobial and antioxidant activities of *Russula delica* Fr. *Food and Chemical Toxicology* 2009, *47*, 2052-2056.

118. Kostić, M.; Ivanov, M.; Fernandes, Â.; Pinela, J.; Calhelha, R.C.; Glamočlija, J.; Barros, L.; Ferreira, I.C.; Soković, M.; Ćirić, A. Antioxidant extracts of three *Russula* species express diverse biological activity. *Molecules* 2020, *25*, 4336.

119. Yoon, K.N.; Lee, T.S. In vitro antioxidant, anti-hyperglycemic, anti-cholinesterase, and inhibition of nitric oxide production activities of methanol and hot water extracts of *Russula rosacea* mushroom. *Journal of Mushroom* 2015, *13*, 1-10.

120. Li, H.; Wang, X.; Xiong, Q.; Yu, Y.; Peng, L. Sulfated modification, characterization, and potential bioactivities of polysaccharide from the fruiting bodies of *Russula virescens*. *International journal of biological macromolecules* 2020, *154*, 1438-1447.

121. Hasnat, M.A.; Pervin, M.; Debnath, T.; Lim, B.O. DNA Protection, Total phenolics and antioxidant potential of the mushroom *Russula virescens*. *Journal of Food Biochemistry* 2014, *38*, 6-17.

122. Leahu, A.; Damian, C.; Oroian, M.; Ropciuc, S. Establishing the antioxidant activity based on chemical composition of wild edible mushrooms. *Food and Environment Safety Journal* 2016, *14*.

123. Volcao, L.M.; Halicki, P.B.; Bilibio, D.; Ramos, D.F.; Bernardi, E.; Da Silva Júnior, F.M.R. Biological activity of aqueous extracts of Southern Brazilian mushrooms. *International Journal of Environmental Health Research* 2021, *31*, 148-159.

124. Piljac-Zegarac, J.; Samec, D.; Piljac, A.; Mesic, A.; Tkalcec, Z. Antioxidant properties of extracts of wild medicinal mushroom species from Croatia. *International Journal of Medicinal Mushrooms* 2011, *13*.

125. Muszyńska, B.; Kała, K.; Radović, J.; Sułkowska-Ziaja, K.; Krakowska, A.; Gdula-Argasińska, J.; Opoka, W.; Kundaković, T. Study of biological activity of Tricholoma equestre fruiting bodies and their safety for human. *European Food Research and Technology* 2018, *244*, 2255-2264.

126. Ragupathi, V.; Stephen, A.; Arivoli, D.; Kumaresan, S. Antioxidant activity of some wild mushrooms from southern Western Ghats, India. *International Journal of Pharmaceutics and Drug Analysis* 2018, 72-79.

127. Gąsecka, M.; Siwulski, M.; Mleczek, M. Evaluation of bioactive compounds content and antioxidant properties of soil‐growing and wood‐growing edible mushrooms. *Journal of Food Processing and Preservation* 2018, *42*, e13386.

128. Yuvali, G.; Onbasli, D. Cytotoxicity of Sarcosphaera crassa and *Tricholoma terreum* extracts on colon cancer cell line (HT-29) in conjunction with their antioxidant properties. *International Journal of Environmental Health Research* 2021, 1-12.

129. Tel, G.; Apaydın, M.; Duru, M.E.; Öztürk, M. Antioxidant and cholinesterase inhibition activities of three *Tricholoma* species with total phenolic and flavonoid contents: the edible mushrooms from Anatolia. *Food Analytical Methods* 2012, *5*, 495-504.

130. Sadi, G.; Kaya, A.; Yalcin, H.A.; Emsen, B.; Kocabas, A.; Kartal, D.I.; Altay, A. Wild edible mushrooms from Turkey as possible anticancer agents on HepG2 cells together with their antioxidant and antimicrobial properties. *International journal of medicinal mushrooms* 2016, *18*.

131. Park, K.M.; Kwon, K.M.; Lee, S.H. Evaluation of the antioxidant activities and tyrosinase inhibitory property from mycelium culture extracts. *Evidence-based complementary and alternative medicine* 2015, *2015*.

132. Ruksiriwanich, W.; Sirithunyalug, J.; Boonpisuttinant, K.; Jantrawut, P. Potent in vitro collagen biosynthesis stimulating and antioxidant activities of edible mushroom Volvariella volvacea aqueous extract. 2014.

133. Nhi, N.; Hung, P. Nutritional composition and antioxidant capacity of several edible mushrooms grown in the Southern Vietnam. 2012.

**Supplementary FIGURE 2** Calibration curve of ABTS radical inhibition % by Trolox.
